# Supplementary material for: Development and validation of a practical score to predict 3-year distant metastatic free survival in nasopharyngeal carcinoma incorporating the number of lymph node regions
Source: PLoS One. 2024 Aug 27;19(8):e0309436. doi: 10.1371/journal.pone.0309436 (PMC11349101; doi:10.1371/journal.pone.0309436)
Supplement: S1 Appendix — (DOCX) [file pone.0309436.s001.docx]

**Supporting information**

Criteria for diagnosis of nodal metastasis and lymph node characteristics in nasopharyngeal carcinoma (NPC) in Magnetic resonance imaging [1].

| Criteria for diagnosis of nodal metastasis | Lateral retropharyngeal LN | MID ≥ 5 mm |
| --- | --- | --- |
|  | Medial retropharyngeal LN | Any size |
|  | Jugulodigatric/diagastric LN | MID ≥ 11 mm |
|  | Other cervical LN | MID ≥ 10 mm |
|  | Other cervical LN | - Any size with central necrosis or ECE  - ≥ 3 contiguous and confluent LN, each MID 8-10 mm |
| Characteristics | Central necrosis | Inhomogeneous signal intensity in LNs (typically high on T2-weighted and low on T1-weighted images) and hypointense non-enhancing areas on postcontrast images. |
|  | Gross ECE | Infiltration into the adjacent fat or muscle |

Abbreviations; LN = lymph node, MRI = Magnetic resonance imaging, MID = minimal axial diameter in the largest plane of an individual node/maximum short-axis diameter, ECE = extracapsular extension

**Criteria for lymph node regions (LRN) [2,3]**

|  |  | Note |
| --- | --- | --- |
| LN region | IA, IB, IIA, IIB, III, IV, VA, VB, VI, VII, RP | 1 level /1 side = 1 region  Except retropharyngeal LN (Bilateral RP was considered as one unit) |
| LN located in the border | LN located at border of 2 regions crossed different axial planes, the status of the node was recorded in both regions. |  |

Abbreviations; LN = lymph node, RP = retropharyngeal LN

**Number of LNR = 1 Number of LNR = 4**


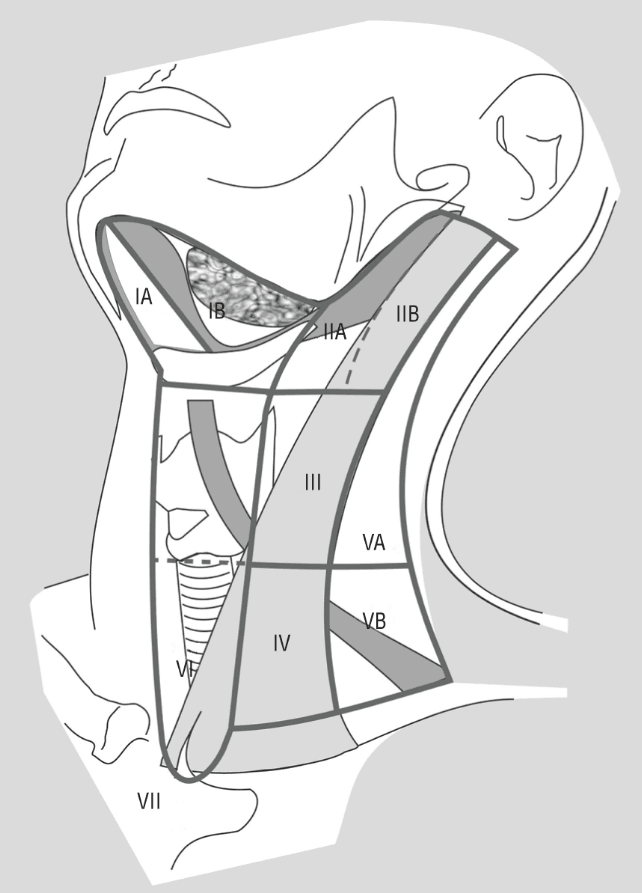

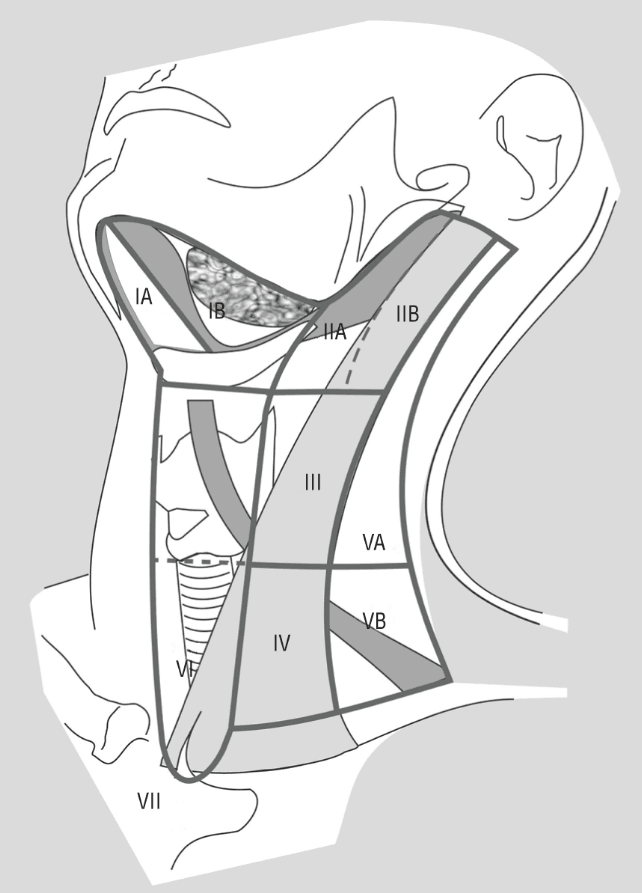


**Details of the treatment**

Nasopharyngeal carcinoma stage II-IVA: concurrent chemoradiation with or without adjuvant chemotherapy

**3.6.1 Radiation treatment**

- All patients were immobilized in the supine position with a tailored head-shoulder thermoplastic mask then a CT simulation was performed.

- MR simulation was performed on every patient and co-registration with the CT images.

- Contouring of clinical target volume (CTV) follows International guidelines for the delineation of the clinical target volumes (CTV) for nasopharyngeal carcinoma [4].

- 2 techniques of radiotherapy

1. Sequential technique: 50Gy in 25 fractions to the PTV-LR followed by 20 Gy in 10 fractions boost to PTV-HR.
2. Simultaneous integrated boost (SIB) technique: 70Gy for PTV-HR at 2.12 Gy/fraction and 56 Gy for PTV-LR at 1.7 Gy/fraction, delivered in 33 fractions.

Volumetric-modulated arc therapy (VMAT) or Intensity-modulated radiation therapy (IMRT) was applied to both techniques.

**3.6.2 Chemotherapy**

**Concurrent chemotherapy regimen:** platinum-based chemotherapy given weekly or tri-weekly.

- Cisplatin 40 mg/m^2^ or carboplatin AUC2 or carboplatin AUC1.5/ paclitaxel 40 mg/m^2^ given weekly.
- Cisplatin 100 mg/m^2^ or carboplatin AUC5 given tri-weekly.

**Adjuvant chemotherapy regimen:** cisplatin/5-fluorouracil or carboplatin/5-fluorouracil at 4-week intervals for 3 cycles

**References**

1. Mao YP, Liang SB, Liu LZ, Chen Y, Sun Y, Tang LL, et al. The N staging system in nasopharyngeal carcinoma with radiation therapy oncology group guidelines for lymph node levels based on magnetic resonance imaging. Clin Cancer Res. 2008;14(22):7497-503.
2. Amin MB, Edge SB, Greene FL, Byrd DR, Brookland RK, Washington MK, et al. AJCC Cancer Staging Manual. 8th ed. New York: Springer International Publishing; 2017.
3. Zhou X, Ou X, Yang Y, Xu T, Shen C, Ding J, et al. Quantitative Metastatic Lymph Node Regions on Magnetic Resonance Imaging Are Superior to AJCC N Classification for the Prognosis of Nasopharyngeal Carcinoma. J Oncol. 2018;2018:9172585.
4. Lee AW, Ng WT, Pan JJ, Poh SS, Ahn YC, AlHussain H, et al. International guideline for the delineation of the clinical target volumes (CTV) for nasopharyngeal carcinoma. Radiother Oncol. 2018 Jan;126(1):25-36.
