# Supplementary material for: Development and validation of a practical score to predict 3-year distant metastatic free survival in nasopharyngeal carcinoma incorporating the number of lymph node regions
Source: PLoS One. 2024 Aug 27;19(8):e0309436. doi: 10.1371/journal.pone.0309436 (PMC11349101; doi:10.1371/journal.pone.0309436)
Supplement: S1 Data — (PDF) [file pone.0309436.s002.pdf]

Study_id	Sex	Age	Age60	WHO_histology	T8	N8	Group_staging	type_conCMT	AdjCMT	EBV_preTx	preEBV2300	No_LNR	LNR3gr	Central_necrosis	ECE	DM_status	Date_DM	Death_status	Date_dead	RTstartdate	Sexscore	T8score	LNR3grscore	EBVscore	Total_score	Riskgroup
1	Female	26	<60	NonkerUndiff SCCA	1	1	2	Cis_weekly	Yes	5160	≥ 2300	2	2-6 regions	No	No	No		No		2/2/2015	0	0	2	1	3	Low_risk
2	Female	48	<60	NonkerUndiff SCCA	3	1	3	Cis_weekly	Yes	0	< 2300	2	2-6 regions	Yes	No	No		No		27/11/2013	0	2	2	0	4	Low_risk
3	Male	51	<60	NonkerUndiff SCCA	3	3	4	Cis_weekly	Yes	8930	≥ 2300	9	7-13 regions	Yes	Yes	Yes	24/4/2015	Yes	16/5/2015	9/2/2015	1	2	3	1	7	High_risk
4	Male	55	<60	NonkerUndiff SCCA	4	1	4	Cis_weekly	Yes	7180	≥ 2300	1	0-1 regions	Yes	No	No		No		19/3/2012	1	2	0	1	4	Low_risk
5	Male	56	<60	NonkerDiffSCCA	4	0	4	Cis_weekly	Yes	0	< 2300	0	0-1 regions	No	No	No		No		3/2/2015	1	2	0	0	3	Low_risk
6	Female	67	≥60	NonkerUndiff SCCA	1	3	4	Cis_weekly	Yes	0	< 2300	12	7-13 regions	No	Yes	No		No		27/3/2012	0	0	3	0	3	Low_risk
7	Male	47	<60	NonkerUndiff SCCA	2	0	2	Cis_weekly	Yes	0	< 2300	0	0-1 regions	No	No	No		No		26/3/2014	1	1	0	0	2	Low_risk
8	Male	44	<60	NonkerUndiff SCCA	3	1	3	Cis_weekly	Yes	7990	≥ 2300	4	2-6 regions	Yes	No	Yes	21/4/2015	Yes	9/1/2020	9/6/2014	1	2	2	1	6	High_risk
9	Male	46	<60	NonkerUndiff SCCA	3	1	3	Cis_weekly	Yes	0	< 2300	1	0-1 regions	Yes	No	No		No		21/2/2011	1	2	0	0	3	Low_risk
10	Male	63	≥60	NonkerUndiff SCCA	2	1	2	Cis_weekly	No	4410	≥ 2300	1	0-1 regions	No	No	No		No		24/2/2015	1	1	0	1	3	Low_risk
11	Female	40	<60	NonkerUndiff SCCA	1	3	4	Cis_weekly	Yes	56000	≥ 2300	10	7-13 regions	Yes	No	No		No		17/2/2015	0	0	3	1	4	Low_risk
12	Male	43	<60	NonkerUndiff SCCA	2	1	2	Cis_weekly	Yes	1760	< 2300	4	2-6 regions	No	No	No		No		27/2/2012	1	1	2	0	4	Low_risk
13	Male	56	<60	NonkerUndiff SCCA	2	0	2	Cis_weekly	Yes	0	< 2300	0	0-1 regions	No	No	No		Yes	8/3/2019	17/4/2012	1	1	0	0	2	Low_risk
14	Female	38	<60	NonkerUndiff SCCA	2	2	3	Cis_weekly	Yes	0	< 2300	7	7-13 regions	Yes	No	No		No		10/3/2014	0	1	3	0	4	Low_risk
15	Male	70	≥60	NonkerDiffSCCA	1	2	3	Cis_weekly	Yes	5620	≥ 2300	6	2-6 regions	Yes	No	Yes	30/3/2017	Yes	7/12/2019	31/3/2014	1	0	2	1	4	Low_risk
16	Male	41	<60	NonkerDiffSCCA	4	2	4	Cis_weekly	Yes	10400	≥ 2300	7	7-13 regions	Yes	No	No		Yes	1/11/2017	18/2/2013	1	2	3	1	7	High_risk
17	Male	49	<60	NonkerDiffSCCA	2	3	4	Cis_weekly	Yes	9690	≥ 2300	9	7-13 regions	Yes	Yes	Yes	6/7/2015	Yes	21/2/2017	27/10/2014	1	1	3	1	6	High_risk
18	Male	48	<60	NonkerUndiff SCCA	1	1	2	Cis_weekly	Yes	1750	< 2300	4	2-6 regions	No	No	No		No		27/8/2012	1	0	2	0	3	Low_risk
19	Male	46	<60	NonkerUndiff SCCA	1	1	2	Cis_weekly	Yes	10800	≥ 2300	1	0-1 regions	No	No	No		No		21/2/2011	1	0	0	1	2	Low_risk
20	Male	39	<60	NonkerUndiff SCCA	1	2	3	Cis_weekly	Yes	0	< 2300	4	2-6 regions	No	No	No		No		2/4/2013	1	0	2	0	3	Low_risk
21	Male	43	<60	NonkerUndiff SCCA	4	1	4	Cis_weekly	Yes	0	< 2300	3	2-6 regions	Yes	No	No		Yes	20/8/2021	22/4/2013	1	2	2	0	5	High_risk
22	Male	62	≥60	NonkerUndiff SCCA	3	1	3	Cis_weekly	Yes	14800	≥ 2300	3	2-6 regions	No	No	Yes	21/8/2014	No		6/3/2013	1	2	2	1	6	High_risk
23	Male	56	<60	NonkerDiffSCCA	3	3	4	Cis_weekly	Yes	893	< 2300	9	7-13 regions	Yes	Yes	No		No		7/4/2015	1	2	3	0	6	High_risk
24	Male	61	≥60	NonkerUndiff SCCA	1	3	4	Cis_weekly	Yes	8020	≥ 2300	10	7-13 regions	Yes	No	No		No		31/3/2014	1	0	3	1	5	High_risk
25	Male	57	<60	NonkerUndiff SCCA	3	2	3	Cis_weekly	Yes	4310	≥ 2300	6	2-6 regions	Yes	No	Yes	26/8/2015	Yes	8/11/2016	20/4/2015	1	2	2	1	6	High_risk
26	Male	50	<60	NonkerUndiff SCCA	4	1	4	Cis_weekly	Yes	11200	≥ 2300	4	2-6 regions	Yes	No	No		No		26/5/2014	1	2	2	1	6	High_risk
27	Male	42	<60	NonkerDiffSCCA	3	1	3	Cis_weekly	Yes	2770	≥ 2300	2	2-6 regions	Yes	No	No		No		27/2/2013	1	2	2	1	6	High_risk
28	Male	63	≥60	NonkerDiffSCCA	4	1	4	Cis_weekly	Yes	0	< 2300	3	2-6 regions	Yes	No	No		No		16/6/2014	1	2	2	0	5	High_risk
29	Female	37	<60	NonkerUndiff SCCA	3	1	3	Cis_weekly	Yes	1800	< 2300	2	2-6 regions	No	No	Yes	13/2/2014	Yes	8/6/2014	15/10/2013	0	2	2	0	4	Low_risk
30	Male	64	≥60	NonkerDiffSCCA	3	3	4	Cis_weekly	Yes	1780	< 2300	9	7-13 regions	No	No	No		No		3/4/2012	1	2	3	0	6	High_risk
31	Female	45	<60	NonkerUndiff SCCA	4	1	4	Cis_weekly	Yes	6880	≥ 2300	3	2-6 regions	No	No	No		No		8/4/2015	0	2	2	1	5	High_risk
32	Female	57	<60	NonkerUndiff SCCA	4	1	4	Cis_weekly	Yes	0	< 2300	3	2-6 regions	Yes	No	No		No		17/4/2014	0	2	2	0	4	Low_risk
33	Female	53	<60	NonkerUndiff SCCA	1	2	3	Cis_weekly	Yes	3540	≥ 2300	6	2-6 regions	Yes	No	No		No		28/10/2013	0	0	2	1	3	Low_risk
34	Female	50	<60	NonkerUndiff SCCA	2	2	3	Cis_weekly	Yes	3510	≥ 2300	8	7-13 regions	No	No	Yes	9/9/2013	Yes	17/11/2015	26/3/2012	0	1	3	1	5	High_risk
35	Male	41	<60	NonkerUndiff SCCA	4	2	4	Cis_weekly	Yes	1000	< 2300	7	7-13 regions	No	No	No		No		29/4/2014	1	2	3	0	6	High_risk
36	Male	70	≥60	NonkerUndiff SCCA	2	1	2	Cis_weekly	Yes	1530	< 2300	2	2-6 regions	Yes	No	No		No		21/4/2014	1	1	2	0	4	Low_risk
37	Female	50	<60	NonkerUndiff SCCA	4	2	4	Cis_weekly	Yes	29300	≥ 2300	3	2-6 regions	Yes	No	No		No		29/4/2014	0	2	2	1	5	High_risk
38	Male	30	<60	NonkerUndiff SCCA	3	2	3	Cis_weekly	Yes	0	< 2300	5	2-6 regions	No	No	Yes	6/6/2017	Yes	25/10/2018	24/6/2014	1	2	2	0	5	High_risk
39	Male	37	<60	NonkerUndiff SCCA	1	1	2	Cis_weekly	Yes	0	< 2300	4	2-6 regions	Yes	No	No		No		5/9/2013	1	0	2	0	3	Low_risk
40	Female	46	<60	NonkerUndiff SCCA	3	1	3	Cis_weekly	Yes	13000	≥ 2300	5	2-6 regions	Yes	Yes	No		No		20/5/2015	0	2	2	1	5	High_risk
41	Male	56	<60	NonkerUndiff SCCA	3	3	4	Cis_weekly	Yes	1150	< 2300	6	2-6 regions	Yes	Yes	No		Yes	23/3/2020	27/5/2013	1	2	2	0	5	High_risk
42	Female	37	<60	NonkerUndiff SCCA	4	3	4	Cis_weekly	Yes	273000	≥ 2300	6	2-6 regions	Yes	No	No		Yes	13/10/2017	13/8/2014	0	2	2	1	5	High_risk
43	Male	55	<60	NonkerUndiff SCCA	1	3	4	Cis_weekly	Yes	42700	≥ 2300	4	2-6 regions	Yes	No	No		No		22/4/2013	1	0	2	1	4	Low_risk
44	Male	45	<60	NonkerDiffSCCA	4	2	4	Cis_weekly	Yes	0	< 2300	5	2-6 regions	Yes	No	No		No		23/6/2015	1	2	2	0	5	High_risk
45	Male	48	<60	NonkerDiffSCCA	4	2	4	Cis_weekly	Yes	0	< 2300	4	2-6 regions	Yes	No	No		Yes	17/10/2019	9/8/2011	1	2	2	0	5	High_risk
46	Male	50	<60	NonkerUndiff SCCA	3	1	3	Cis_weekly	Yes	2240	< 2300	1	0-1 regions	No	No	No		Yes	12/6/2022	10/6/2014	1	2	0	0	3	Low_risk
47	Male	51	<60	NonkerUndiff SCCA	2	1	2	Cis_weekly	Yes	0	< 2300	1	0-1 regions	No	No	No		No		18/4/2012	1	1	0	0	2	Low_risk
48	Female	38	<60	NonkerUndiff SCCA	3	2	3	Cis_weekly	Yes	6390	≥ 2300	5	2-6 regions	Yes	No	Yes	21/12/2015	Yes	5/8/2018	11/4/2012	0	2	2	1	5	High_risk
49	Female	52	<60	NonkerUndiff SCCA	3	1	3	Cis_weekly	Yes	0	< 2300	1	0-1 regions	No	No	No		No		11/6/2013	0	2	0	0	2	Low_risk
50	Female	55	<60	NonkerUndiff SCCA	2	0	2	Cis_weekly	Yes	0	< 2300	0	0-1 regions	No	No	No		No		28/5/2013	0	1	0	0	1	Low_risk
51	Female	43	<60	NonkerUndiff SCCA	2	3	4	Cis_weekly	Yes	0	< 2300	6	2-6 regions	No	No	No		No		30/4/2012	0	1	2	0	3	Low_risk
52	Male	44	<60	NonkerUndiff SCCA	2	1	2	Cis_weekly	Yes	10700	≥ 2300	2	2-6 regions	No	No	No		No		30/4/2012	1	1	2	1	5	High_risk
53	Female	38	<60	NonkerUndiff SCCA	2	3	4	Cis_weekly	Yes	14400	≥ 2300	8	7-13 regions	No	No	No		No		8/5/2013	0	1	3	1	5	High_risk
54	Female	62	≥60	NonkerUndiff SCCA	1	1	2	Cis_weekly	Yes	0	< 2300	1	0-1 regions	No	No	No		No		29/5/2012	0	0	0	0	0	Low_risk
55	Male	39	<60	NonkerUndiff SCCA	4	3	4	Cis_weekly	Yes	6140	≥ 2300	9	7-13 regions	Yes	No	Yes	16/6/2016	Yes	14/5/2017	15/6/2015	1	2	3	1	7	High_risk
56	Female	52	<60	NonkerUndiff SCCA	2	2	3	Cis_weekly	Yes	7860	≥ 2300	7	7-13 regions	Yes	No	Yes	9/9/2013	Yes	17/1/2019	27/12/2011	0	1	3	1	5	High_risk
57	Male	63	≥60	NonkerUndiff SCCA	2	2	3	Cis_weekly	Yes	45700	≥ 2300	7	7-13 regions	Yes	No	Yes	16/3/2017	Yes	6/7/2019	18/6/2015	1	1	3	1	6	High_risk
58	Male	53	<60	NonkerDiffSCCA	1	1	2	Cis_weekly	Yes	0	< 2300	2	2-6 regions	No	No	No		No		28/4/2014	1	0	2	0	3	Low_risk
59	Male	52	<60	NonkerUndiff SCCA	4	1	4	Cis_weekly	Yes	13700	≥ 2300	1	0-1 regions	No	No	No		Yes	25/4/2013	13/6/2011	1	2	0	1	4	Low_risk
60	Male	39	<60	NonkerUndiff SCCA	4	1	4	Cis_weekly	Yes	3150	≥ 2300	2	2-6 regions	Yes	No	No		No		14/6/2012	1	2	2	1	6	High_risk
61	Female	50	<60	NonkerUndiff SCCA	1	2	3	Cis_weekly	Yes	2350	≥ 2300	6	2-6 regions	Yes	No	No		No		6/8/2012	0	0	2	1	3	Low_risk
62	Male	50	<60	NonkerUndiff SCCA	4	3	4	Cis_triweekly	Yes	46100	≥ 2300	9	7-13 regions	Yes	Yes	Yes	4/4/2013	Yes	30/5/2017	18/6/2012	1	2	3	1	7	High_risk
63	Male	52	<60	NonkerUndiff SCCA	3	2	3	Cis_weekly	Yes	48000	≥ 2300	10	7-13 regions	Yes	Yes	Yes	1/12/2014	No		24/7/2012	1	2	3	1	7	High_risk
64	Male	47	<60	NonkerUndiff SCCA	2	1	2	Cis_weekly	Yes	0	< 2300	1	0-1 regions	No	No	No		No		6/6/2011	1	1	0	0	2	Low_risk
65	Male	53	<60	NonkerUndiff SCCA	3	1	3	Cis_triweekly	Yes	0	< 2300	1	0-1 regions	No	No	No		No		21/4/2014	1	2	0	0	3	Low_risk
66	Male	44	<60	NonkerUndiff SCCA	2	1	2	Cis_weekly	Yes	2000	< 2300	2	2-6 regions	Yes	No	No		No		22/4/2013	1	1	2	0	4	Low_risk
67	Male	47	<60	NonkerUndiff SCCA	2	2	3	Cis_weekly	Yes	0	< 2300	3	2-6 regions	Yes	No	No		No		2/7/2012	1	1	2	0	4	Low_risk
68	Male	43	<60	NonkerUndiff SCCA	2	2	3	Cis_weekly	Yes	0	< 2300	3	2-6 regions	No	No	No		No		29/7/2014	1	1	2	0	4	Low_risk
69	Male	52	<60	NonkerUndiff SCCA	2	1	2	Cis_weekly	Yes	0	< 2300	1	0-1 regions	No	No	No		No		5/8/2013	1	1	0	0	2	Low_risk
70	Male	43	<60	NonkerUndiff SCCA	3	1	3	Cis_weekly	Yes	0	< 2300	3	2-6 regions	No	No	No		No		24/6/2013	1	2	2	0	5	High_risk
71	Male	54	<60	NonkerUndiff SCCA	1	3	4	Cis_weekly	Yes	37400	≥ 2300	8	7-13 regions	Yes	No	Yes	8/6/2017	No		6/7/2015	1	0	3	1	5	High_risk
72	Male	41	<60	NonkerUndiff SCCA	2	2	3	Cis_weekly	Yes	7520	≥ 2300	5	2-6 regions	No	No	No		No		18/6/2012	1	1	2	1	5	High_risk
73	Female	32	<60	NonkerUndiff SCCA	3	1	3	Cis_weekly	Yes	11300	≥ 2300	3	2-6 regions	No	No	No		No		1/7/2013	0	2	2	1	5	High_risk
74	Male	26	<60	NonkerUndiff SCCA	2	1	2	Cis_weekly	Yes	2640	≥ 2300	3	2-6 regions	Yes	No	Yes	1/11/2013	Yes	27/7/2016	16/7/2012	1	1	2	1	5	High_risk
75	Male	73	≥60	NonkerUndiff SCCA	3	2	3	Cis_weekly	Yes	23400	≥ 2300	4	2-6 regions	Yes	No	No		Yes	17/4/2014	9/8/2013	1	2	2	1	6	High_risk
76	Male	56	<60	NonkerUndiff SCCA	1	3	4	Cis_weekly	Yes	829	< 2300	5	2-6 regions	No	No	No		No		25/7/2012	1	0	2	0	3	Low_risk
77	Male	68	≥60	NonkerDiffSCCA	4	2	4	Cis_weekly	Yes	0	< 2300	7	7-13 regions	Yes	Yes	Yes	23/12/2014	Yes	10/1/2016	2/7/2012	1	2	3	0	6	High_risk
78	Male	40	<60	NonkerUndiff SCCA	1	1	2	Cis_weekly	Yes	0	< 2300	2	2-6 regions	No	No	No		No		25/5/2015	1	0	2	0	3	Low_risk
79	Male	47	<60	NonkerDiffSCCA	2	2	3	Cis_weekly	Yes	0	< 2300	6	2-6 regions	No	No	No		No		8/7/2013	1	1	2	0	4	Low_risk
80	Male	47	<60	NonkerUndiff SCCA	3	2	3	Cis_weekly	Yes	3270	≥ 2300	7	7-13 regions	No	No	No		No		27/7/2015	1	2	3	1	7	High_risk
81	Male	47	<60	NonkerUndiff SCCA	3	1	3	Cis_weekly	Yes	1510	< 2300	1	0-1 regions	No	No	No		No		20/7/2015	1	2	0	0	3	Low_risk
82	Male	54	<60	NonkerUndiff SCCA	2	2	3	Cis_weekly	Yes	4350	≥ 2300	7	7-13 regions	Yes	No	No		No		23/8/2012	1	1	3	1	6	High_risk
83	Male	53	<60	NonkerUndiff SCCA	2	1	2	Cis_weekly	Yes	4130	≥ 2300	3	2-6 regions	No	No	No		No		18/7/2013	1	1	2	1	5	High_risk
84	Female	44	<60	NonkerUndiff SCCA	2	2	3	Cis_weekly	Yes	3960	≥ 2300	9	7-13 regions	Yes	No	No		No		23/8/2012	0	1	3	1	5	High_risk
85	Male	34	<60	NonkerUndiff SCCA	1	2	3	Cis_weekly	Yes	0	< 2300	6	2-6 regions	Yes	No	No		No		29/8/2013	1	0	2	0	3	Low_risk
86	Male	60	≥60	NonkerUndiff SCCA	2	1	2	Cis_weekly	Yes	12200	≥ 2300	4	2-6 regions	Yes	No	Yes	17/2/2015	Yes	26/6/2016	29/7/2013	1	1	2	1	5	High_risk
87	Male	54	<60	NonkerDiffSCCA	2	1	2	Cis_weekly	Yes	0	< 2300	3	2-6 regions	No	No	No		No		24/7/2015	1	1	2	0	4	Low_risk
88	Female	32	<60	NonkerUndiff SCCA	2	1	2	Cis_weekly	Yes	0	< 2300	1	0-1 regions	No	No	No		Yes	1/7/2021	5/8/2013	0	1	0	0	1	Low_risk
89	Female	63	≥60	NonkerUndiff SCCA	2	1	2	Cis_weekly	Yes	0	< 2300	1	0-1 regions	No	No	Yes	19/6/2018	No		19/5/2014	0	1	0	0	1	Low_risk
90	Male	45	<60	NonkerDiffSCCA	2	0	2	Cis_weekly	Yes	0	< 2300	0	0-1 regions	No	No	No		No		9/8/2013	1	1	0	0	2	Low_risk
91	Male	53	<60	NonkerUndiff SCCA	1	1	2	Cis_weekly	Yes	0	< 2300	1	0-1 regions	No	No	No		No		19/8/2013	1	0	0	0	1	Low_risk
92	Male	27	<60	NonkerDiffSCCA	1	1	2	Cis_weekly	Yes	0	< 2300	2	2-6 regions	Yes	No	No		No		2/12/2013	1	0	2	0	3	Low_risk
93	Male	40	<60	NonkerUndiff SCCA	3	1	3	Cis_weekly	Yes	15900	≥ 2300	2	2-6 regions	Yes	No	No		Yes	13/10/2013	7/2/2012	1	2	2	1	6	High_risk
94	Male	44	<60	NonkerUndiff SCCA	3	1	3	Cis_weekly	Yes	0	< 2300	5	2-6 regions	Yes	No	No		No		5/8/2013	1	2	2	0	5	High_risk
95	Male	44	<60	NonkerUndiff SCCA	1	1	2	Cis_triweekly	Yes	0	< 2300	2	2-6 regions	No	No	No		No		19/8/2013	1	0	2	0	3	Low_risk
96	Male	66	≥60	NonkerUndiff SCCA	2	1	2	Cis_weekly	Yes	12800	≥ 2300	2	2-6 regions	No	No	No		No		25/9/2012	1	1	2	1	5	High_risk
97	Male	43	<60	NonkerUndiff SCCA	2	3	4	Carbo_weekly	Yes	1980	< 2300	6	2-6 regions	Yes	Yes	No		No		3/10/2013	1	1	2	0	4	Low_risk
98	Male	61	≥60	NonkerUndiff SCCA	1	1	2	Cis_weekly	Yes	2400	≥ 2300	3	2-6 regions	No	No	No		No		2/9/2013	1	0	2	1	4	Low_risk
99	Male	36	<60	NonkerUndiff SCCA	1	1	2	Cis_weekly	Yes	0	< 2300	3	2-6 regions	No	No	No		No		14/9/2015	1	0	2	0	3	Low_risk
100	Female	40	<60	NonkerUndiff SCCA	1	3	4	Cis_weekly	Yes	10400	≥ 2300	5	2-6 regions	Yes	No	No		No		25/9/2013	0	0	2	1	3	Low_risk
101	Male	62	≥60	NonkerUndiff SCCA	1	3	4	Cis_weekly	Yes	28500	≥ 2300	7	7-13 regions	No	No	No		Yes	21/4/2019	16/9/2013	1	0	3	1	5	High_risk
102	Female	58	<60	NonkerDiffSCCA	3	2	3	Cis_weekly	Yes	0	< 2300	4	2-6 regions	No	Yes	No		No		14/9/2015	0	2	2	0	4	Low_risk
103	Male	45	<60	NonkerUndiff SCCA	1	1	2	Cis_weekly	Yes	8210	≥ 2300	4	2-6 regions	No	No	No		No		16/9/2013	1	0	2	1	4	Low_risk
104	Male	55	<60	NonkerUndiff SCCA	3	2	3	Cis_weekly	No	2010	< 2300	5	2-6 regions	No	Yes	No		No		8/10/2012	1	2	2	0	5	High_risk
105	Male	65	≥60	NonkerUndiff SCCA	1	1	2	Cis_weekly	Yes	3380	≥ 2300	3	2-6 regions	No	No	No		No		23/4/2012	1	0	2	1	4	Low_risk
106	Male	39	<60	NonkerDiffSCCA	4	2	4	Cis_weekly	Yes	6020	≥ 2300	6	2-6 regions	No	No	No		No		25/3/2014	1	2	2	1	6	High_risk
107	Female	33	<60	NonkerUndiff SCCA	2	1	2	Cis_weekly	Yes	2520	≥ 2300	1	0-1 regions	No	No	No		No		23/9/2013	0	1	0	1	2	Low_risk
108	Male	60	≥60	NonkerUndiff SCCA	3	1	3	Cis_weekly	Yes	262000	≥ 2300	4	2-6 regions	Yes	Yes	Yes	31/5/2017	Yes	20/4/2021	21/9/2015	1	2	2	1	6	High_risk
109	Male	27	<60	NonkerUndiff SCCA	4	1	4	Cis_weekly	Yes	0	< 2300	3	2-6 regions	No	No	No		No		5/11/2013	1	2	2	0	5	High_risk
110	Male	30	<60	NonkerUndiff SCCA	4	2	4	Cis_weekly	Yes	85700	≥ 2300	4	2-6 regions	Yes	No	Yes	17/4/2013	Yes	30/5/2014	4/10/2012	1	2	2	1	6	High_risk
111	Male	60	≥60	NonkerUndiff SCCA	3	2	3	Cis_weekly	Yes	47800	≥ 2300	4	2-6 regions	No	No	No		Yes	31/1/2016	30/10/2012	1	2	2	1	6	High_risk
112	Male	53	<60	NonkerUndiff SCCA	3	1	3	Cis_weekly	Yes	0	< 2300	1	0-1 regions	No	No	No		No		29/7/2014	1	2	0	0	3	Low_risk
113	Male	45	<60	NonkerUndiff SCCA	2	2	3	Cis_weekly	Yes	817	< 2300	4	2-6 regions	No	No	No		No		13/11/2012	1	1	2	0	4	Low_risk
114	Male	42	<60	NonkerUndiff SCCA	4	3	4	Cis_weekly	Yes	2350	≥ 2300	9	7-13 regions	Yes	Yes	Yes	21/2/2013	Yes	10/5/2013	21/11/2011	1	2	3	1	7	High_risk
115	Male	72	≥60	NonkerUndiff SCCA	4	3	4	Cis_weekly	Yes	6760	≥ 2300	6	2-6 regions	No	Yes	No		Yes	29/7/2015	5/8/2014	1	2	2	1	6	High_risk
116	Male	74	≥60	NonkerUndiff SCCA	4	1	4	Carbo_weekly	Yes	6860	≥ 2300	2	2-6 regions	No	No	No		Yes	16/7/2014	13/11/2013	1	2	2	1	6	High_risk
117	Male	45	<60	NonkerDiffSCCA	4	1	4	Cis_weekly	Yes	0	< 2300	1	0-1 regions	No	No	No		No		23/12/2013	1	2	0	0	3	Low_risk
118	Female	61	≥60	NonkerUndiff SCCA	1	2	3	Cis_weekly	Yes	11000	≥ 2300	5	2-6 regions	No	No	No		No		28/11/2013	0	0	2	1	3	Low_risk
119	Male	58	<60	NonkerUndiff SCCA	3	1	3	Cis_weekly	Yes	8280	≥ 2300	3	2-6 regions	Yes	No	No		Yes	11/5/2014	5/11/2013	1	2	2	1	6	High_risk
120	Male	68	≥60	NonkerUndiff SCCA	4	2	4	Cis_weekly	Unknown	0	< 2300	5	2-6 regions	Yes	No	No		Yes	17/9/2014	17/10/2011	1	2	2	0	5	High_risk
121	Male	51	<60	NonkerUndiff SCCA	3	1	3	Cis_weekly	Yes	10600	≥ 2300	3	2-6 regions	Yes	Yes	No		No		30/6/2014	1	2	2	1	6	High_risk
122	Male	70	≥60	NonkerUndiff SCCA	3	1	3	Cis_weekly	Yes	19300	≥ 2300	3	2-6 regions	Yes	No	No		No		3/6/2014	1	2	2	1	6	High_risk
123	Male	47	<60	NonkerUndiff SCCA	2	1	2	Cis_weekly	Yes	0	< 2300	3	2-6 regions	No	No	Yes	31/1/2017	No		29/12/2011	1	1	2	0	4	Low_risk
124	Female	41	<60	NonkerDiffSCCA	3	2	3	Cis_weekly	Yes	0	< 2300	6	2-6 regions	No	No	No		No		22/11/2011	0	2	2	0	4	Low_risk
125	Male	53	<60	NonkerDiffSCCA	2	1	2	Cis_weekly	Yes	0	< 2300	1	0-1 regions	No	No	No		No		15/9/2014	1	1	0	0	2	Low_risk
126	Male	46	<60	NonkerUndiff SCCA	2	1	2	Cis_weekly	Yes	0	< 2300	1	0-1 regions	No	No	No		No		20/11/2012	1	1	0	0	2	Low_risk
127	Male	60	≥60	NonkerUndiff SCCA	3	3	4	Cis_weekly	Yes	18600	≥ 2300	9	7-13 regions	Yes	Yes	No		No		10/10/2013	1	2	3	1	7	High_risk
128	Male	62	≥60	NonkerUndiff SCCA	3	3	4	Carbo_weekly	Yes	0	< 2300	11	7-13 regions	Yes	No	No		Yes	19/4/2013	26/12/2012	1	2	3	0	6	High_risk
129	Female	39	<60	NonkerUndiff SCCA	1	1	2	Cis_weekly	Yes	0	< 2300	1	0-1 regions	No	No	No		No		24/11/2015	0	0	0	0	0	Low_risk
130	Female	43	<60	NonkerUndiff SCCA	4	1	4	Cis_weekly	Yes	0	< 2300	1	0-1 regions	No	No	No		No		13/12/2011	0	2	0	0	2	Low_risk
131	Male	58	<60	NonkerDiffSCCA	3	3	4	Cis_weekly	Yes	281000	≥ 2300	10	7-13 regions	Yes	No	No		Yes	3/1/2015	9/1/2012	1	2	3	1	7	High_risk
132	Male	33	<60	NonkerUndiff SCCA	2	3	4	Cis_triweekly	Yes	11000	≥ 2300	10	7-13 regions	No	Yes	No		No		15/1/2013	1	1	3	1	6	High_risk
133	Male	51	<60	NonkerDiffSCCA	2	1	2	Cis_weekly	Yes	0	< 2300	1	0-1 regions	No	No	No		No		25/11/2013	1	1	0	0	2	Low_risk
134	Male	49	<60	NonkerDiffSCCA	4	2	4	Carbo_weekly	Yes	7320	≥ 2300	7	7-13 regions	No	No	No		Yes	6/11/2014	24/1/2014	1	2	3	1	7	High_risk
135	Male	51	<60	NonkerUndiff SCCA	4	1	4	Cis_weekly	Yes	37600	≥ 2300	4	2-6 regions	Yes	No	No		No		7/12/2011	1	2	2	1	6	High_risk
136	Male	45	<60	NonkerUndiff SCCA	1	3	4	Cis_weekly	Unknown	28800	≥ 2300	6	2-6 regions	Yes	No	Yes	9/11/2015	Yes	9/11/2015	6/12/2011	1	0	2	1	4	Low_risk
137	Male	61	≥60	NonkerUndiff SCCA	2	1	2	Cis_weekly	Yes	0	< 2300	2	2-6 regions	No	No	No		No		12/11/2012	1	1	2	0	4	Low_risk
138	Male	68	≥60	NonkerUndiff SCCA	2	1	2	Carbo_weekly	Yes	1260	< 2300	1	0-1 regions	No	No	No		No		5/7/2016	1	1	0	0	2	Low_risk
139	Female	53	<60	NonkerUndiff SCCA	1	1	2	Cis_weekly	Yes	3250	≥ 2300	4	2-6 regions	No	No	No		No		17/3/2014	0	0	2	1	3	Low_risk
140	Male	40	<60	NonkerDiffSCCA	1	1	2	Cis_weekly	Yes	0	< 2300	1	0-1 regions	No	No	No		No		17/1/2012	1	0	0	0	1	Low_risk
141	Male	46	<60	NonkerUndiff SCCA	3	1	3	Cis_weekly	Unknown	5130	≥ 2300	3	2-6 regions	No	No	No		No		19/2/2014	1	2	2	1	6	High_risk
142	Male	45	<60	NonkerUndiff SCCA	4	3	4	Cis_weekly	Yes	40900	≥ 2300	9	7-13 regions	Yes	Yes	Yes	12/3/2015	Yes	21/5/2018	20/1/2014	1	2	3	1	7	High_risk
143	Male	45	<60	NonkerUndiff SCCA	2	3	4	Cis_weekly	Yes	5340	≥ 2300	8	7-13 regions	Yes	No	No		No		26/11/2012	1	1	3	1	6	High_risk
144	Female	51	<60	NonkerDiffSCCA	3	2	3	Cis_weekly	Yes	2410	≥ 2300	5	2-6 regions	No	No	Yes	7/11/2013	Yes	13/7/2014	23/1/2012	0	2	2	1	5	High_risk
145	Male	18	<60	NonkerUndiff SCCA	4	1	4	Cis_weekly	Yes	0	< 2300	2	2-6 regions	No	No	Yes	21/1/2015	Yes	9/6/2019	18/2/2014	1	2	2	0	5	High_risk
146	Female	35	<60	NonkerUndiff SCCA	3	1	3	Cis_weekly	Yes	0	< 2300	1	0-1 regions	No	No	No		No		19/2/2013	0	2	0	0	2	Low_risk
147	Male	52	<60	NonkerUndiff SCCA	3	1	3	Cis_weekly	Yes	0	< 2300	1	0-1 regions	Yes	No	Yes	16/4/2015	No		24/12/2012	1	2	0	0	3	Low_risk
148	Male	44	<60	NonkerUndiff SCCA	3	2	3	Cis_weekly	Yes	18200	≥ 2300	5	2-6 regions	Yes	No	No		No		23/2/2015	1	2	2	1	6	High_risk
149	Male	72	≥60	NonkerUndiff SCCA	2	1	2	Cis_weekly	Yes	0	< 2300	4	2-6 regions	Yes	Yes	No		No		27/11/2013	1	1	2	0	4	Low_risk
150	Female	18	<60	NonkerUndiff SCCA	3	2	3	Cis_weekly	Yes	2590	≥ 2300	4	2-6 regions	No	No	No		No		17/6/2014	0	2	2	1	5	High_risk
151	Male	43	<60	NonkerUndiff SCCA	2	0	2	Cis_weekly	Unknown	0	< 2300	0	0-1 regions	No	No	No		No		13/8/2014	1	1	0	0	2	Low_risk
152	Male	38	<60	NonkerUndiff SCCA	1	1	2	Cis_weekly	Yes	0	< 2300	1	0-1 regions	No	No	No		No		7/7/2014	1	0	0	0	1	Low_risk
153	Male	42	<60	NonkerUndiff SCCA	3	1	3	Cis_weekly	Yes	24100	≥ 2300	1	0-1 regions	No	No	Yes	19/10/2017	Yes	16/12/2019	29/7/2014	1	2	0	1	4	Low_risk
154	Male	40	<60	NonkerUndiff SCCA	3	1	3	Cis_weekly	Yes	0	< 2300	1	0-1 regions	No	No	No		No		18/8/2014	1	2	0	0	3	Low_risk
155	Male	44	<60	NonkerUndiff SCCA	2	1	2	Cis_weekly	Yes	0	< 2300	1	0-1 regions	No	No	No		Yes	27/1/2019	21/7/2014	1	1	0	0	2	Low_risk
156	Male	47	<60	NonkerUndiff SCCA	1	1	2	Cis_weekly	Yes	0	< 2300	4	2-6 regions	No	No	No		No		2/10/2014	1	0	2	0	3	Low_risk
157	Female	41	<60	NonkerUndiff SCCA	2	1	2	Cis_weekly	Yes	0	< 2300	4	2-6 regions	No	No	No		No		1/9/2014	0	1	2	0	3	Low_risk
158	Male	49	<60	NonkerUndiff SCCA	3	1	3	Cis_weekly	Yes	0	< 2300	1	0-1 regions	No	No	No		Yes	13/12/2020	15/6/2015	1	2	0	0	3	Low_risk
159	Male	50	<60	NonkerUndiff SCCA	1	1	2	Cis_weekly	Yes	0	< 2300	5	2-6 regions	No	No	No		No		30/9/2014	1	0	2	0	3	Low_risk
160	Male	51	<60	NonkerUndiff SCCA	4	1	4	Cis_weekly	Yes	0	< 2300	4	2-6 regions	No	No	Yes	22/10/2015	Yes	7/6/2016	31/10/2014	1	2	2	0	5	High_risk
161	Male	44	<60	NonkerUndiff SCCA	3	3	4	Carbo_weekly	Yes	18000	≥ 2300	9	7-13 regions	No	No	Yes	8/1/2016	Yes	3/5/2016	17/11/2014	1	2	3	1	7	High_risk
162	Female	63	≥60	NonkerUndiff SCCA	3	2	3	Cis_weekly	Unknown	162000	≥ 2300	7	7-13 regions	Yes	No	Yes	6/1/2017	Yes	6/1/2017	5/11/2014	0	2	3	1	6	High_risk
163	Male	56	<60	NonkerDiffSCCA	1	2	3	Cis_weekly	Yes	2100	< 2300	4	2-6 regions	Yes	No	No		No		20/10/2014	1	0	2	0	3	Low_risk
164	Male	62	≥60	NonkerUndiff SCCA	1	1	2	Cis_weekly	Yes	0	< 2300	2	2-6 regions	No	No	No		Yes	1/6/2021	30/6/2015	1	0	2	0	3	Low_risk
165	Male	65	≥60	NonkerDiffSCCA	3	1	3	Cis_weekly	Yes	6720	≥ 2300	2	2-6 regions	No	No	No		Yes	4/12/2016	5/1/2015	1	2	2	1	6	High_risk
166	Male	68	≥60	NonkerUndiff SCCA	4	1	4	Carbo_weekly	Yes	19600	≥ 2300	3	2-6 regions	No	No	Yes	19/1/2016	Yes	20/9/2017	9/2/2015	1	2	2	1	6	High_risk
167	Female	59	<60	NonkerUndiff SCCA	3	1	3	Cis_weekly	Yes	9720	≥ 2300	4	2-6 regions	No	No	No		No		19/1/2015	0	2	2	1	5	High_risk

168	Female	56	<60	NonkerUndiff SCCA	4	2	4	Cis triweekly	No	8379	≥ 2300	4	2-6 regions	Yes	No	Yes	21/6/2017	No		17/10/2015	0	2	2	1	5	High risk
169	Female	73	≥60	NonkerUndiff SCCA	1	1	2	Cis weekly	No	0	< 2300	3	2-6 regions	Yes	No	No		No		24/3/2014	0	0	2	0	2	Low risk
170	Male	42	<60	NonkerUndiff SCCA	4	1	4	Cis triweekly	Yes	1800	< 2300	2	2-6 regions	No	No	No		Yes	14/8/2021	31/8/2017	1	2	2	0	5	High risk
171	Female	30	<60	NonkerDiffSCCA	4	2	4	Cis triweekly	Yes	2520	≥ 2300	6	2-6 regions	No	No	No		No		11/12/2013	0	2	2	1	5	High risk
172	Male	31	<60	NonkerUndiff SCCA	4	1	4	Cis weekly	No	3660	≥ 2300	3	2-6 regions	Yes	No	No		No		19/8/2015	1	2	2	1	6	High risk
173	Male	56	<60	NonkerUndiff SCCA	4	1	4	Unknown	No	0	< 2300	4	2-6 regions	No	No	No		No		6/12/2018	1	2	2	0	5	High risk
174	Female	37	<60	NonkerUndiff SCCA	3	2	3	Cis triweekly	Yes	0	< 2300	5	2-6 regions	Yes	Yes	No		No		10/3/2015	0	2	2	0	4	Low risk
175	Male	55	<60	NonkerUndiff SCCA	4	1	4	Cis weekly	Yes	135000	≥ 2300	3	2-6 regions	No	Yes	No		No		9/4/2017	1	2	2	1	6	High risk
176	Female	52	<60	NonkerUndiff SCCA	3	3	4	Cis triweekly	Yes	0	< 2300	5	2-6 regions	Yes	Yes	No		Yes	23/2/2018	2/2/2017	0	2	2	0	4	Low risk
177	Male	57	<60	NonkerUndiff SCCA	2	2	3	Cis triweekly	Yes	66780	≥ 2300	5	2-6 regions	Yes	No	No		No		21/1/2016	1	1	2	1	5	High risk
178	Male	59	<60	NonkerUndiff SCCA	3	1	3	Cis weekly	Yes	2943	≥ 2300	2	2-6 regions	No	No	No		Yes	12/3/2016	21/12/2015	1	2	2	1	6	High risk
179	Male	50	<60	NonkerUndiff SCCA	3	2	3	Cis weekly	Yes	0	< 2300	3	2-6 regions	Yes	No	No		No		7/4/2015	1	2	2	0	5	High risk
180	Male	38	<60	NonkerUndiff SCCA	1	1	2	Cis weekly	No	0	< 2300	1	0-1 regions	No	No	No		No		3/1/2018	1	0	0	0	1	Low risk
181	Male	59	<60	NonkerUndiff SCCA	3	2	3	Carbo weekly	Yes	2451	≥ 2300	6	2-6 regions	Yes	No	No		No		25/1/2017	1	2	2	1	6	High risk
182	Male	58	<60	NonkerUndiff SCCA	1	1	2	Cis weekly	Yes	3950	≥ 2300	3	2-6 regions	No	No	No		No		20/5/2015	1	0	2	1	4	Low risk
183	Male	54	<60	NonkerUndiff SCCA	2	0	2	Cis triweekly	Yes	0	< 2300	0	0-1 regions	No	No	No		No		20/7/2010	1	1	0	0	2	Low risk
184	Male	60	≥60	NonkerDiffSCCA	2	3	4	Cis weekly	Yes	95300	≥ 2300	7	7-13 regions	Yes	No	Yes	5/1/2014	Yes	13/1/2014	14/8/2013	1	1	3	1	6	High risk
185	Female	33	<60	NonkerUndiff SCCA	2	1	2	Cis weekly	Yes	3400	≥ 2300	1	0-1 regions	No	No	No		No		24/8/2015	0	1	0	1	2	Low risk
186	Female	47	<60	NonkerUndiff SCCA	1	1	2	Cis weekly	Yes	0	< 2300	1	0-1 regions	No	No	No		No		17/12/2012	0	0	0	0	0	Low risk
187	Male	67	≥60	NonkerUndiff SCCA	2	1	2	Cis weekly	Unknown	0	< 2300	1	0-1 regions	No	No	No		Yes	18/1/2016	11/3/2013	1	1	0	0	2	Low risk
188	Male	42	<60	NonkerUndiff SCCA	4	1	4	Unknown	Unknown	73900	≥ 2300	1	0-1 regions	No	No	No		Yes	12/11/2020	26/8/2014	1	2	0	1	4	Low risk
189	Male	50	<60	NonkerUndiff SCCA	1	1	2	Cis weekly	Yes	370	< 2300	2	2-6 regions	No	No	No		No		15/1/2018	1	0	2	0	3	Low risk
190	Female	53	<60	NonkerUndiff SCCA	3	1	3	Cis weekly	No	0	< 2300	1	0-1 regions	No	No	No		No		1/4/2019	0	2	0	0	2	Low risk
191	Male	20	<60	NonkerUndiff SCCA	3	1	3	Cis triweekly	Yes	2780	≥ 2300	3	2-6 regions	No	No	No		No		14/1/2019	1	2	2	1	6	High risk
192	Female	47	<60	NonkerUndiff SCCA	2	1	2	Cis weekly	No	0	< 2300	1	0-1 regions	No	No	No		No		12/6/2019	0	1	0	0	1	Low risk
193	Female	39	<60	NonkerUndiff SCCA	3	1	3	Cis weekly	No	0	< 2300	2	2-6 regions	No	No	No		No		28/1/2019	0	2	2	0	4	Low risk
194	Male	71	≥60	NonkerUndiff SCCA	2	2	3	Cis weekly	Yes	0	< 2300	4	2-6 regions	Yes	No	No		No		15/7/2014	1	1	2	0	4	Low risk
195	Male	45	<60	NonkerUndiff SCCA	1	1	2	Cis weekly	Yes	0	< 2300	1	0-1 regions	No	No	No		No		15/7/2014	1	0	0	0	1	Low risk
196	Male	33	<60	NonkerUndiff SCCA	3	2	3	Unknown	No	0	< 2300	3	2-6 regions	Yes	No	No		Yes	6/7/2021	15/10/2014	1	2	2	0	5	High risk
197	Female	53	<60	NonkerDiffSCCA	1	1	2	Cis weekly	No	0	< 2300	1	0-1 regions	No	No	No		No		3/11/2014	0	0	0	0	0	Low risk
198	Male	29	<60	NonkerUndiff SCCA	1	3	4	Cis triweekly	Yes	0	< 2300	6	2-6 regions	Yes	No	No		No		18/9/2017	1	0	2	0	3	Low risk
199	Female	67	≥60	NonkerUndiff SCCA	3	1	3	Cis weekly	No	91800	≥ 2300	3	2-6 regions	Yes	No	No		Yes	12/2/2018	31/8/2016	0	2	2	1	5	High risk
200	Male	46	<60	NonkerUndiff SCCA	2	1	2	Cis weekly	Yes	0	< 2300	1	0-1 regions	No	No	No		No		20/12/2019	1	1	0	0	2	Low risk
201	Female	58	<60	NonkerUndiff SCCA	3	2	3	Cis weekly	Yes	2900	≥ 2300	3	2-6 regions	Yes	No	No		No		23/3/2015	0	2	2	1	5	High risk
202	Male	61	≥60	NonkerUndiff SCCA	3	3	4	Cis weekly	No	21780	≥ 2300	6	2-6 regions	Yes	No	Yes	5/2/2018	No		20/4/2016	1	2	2	1	6	High risk
203	Male	56	<60	NonkerUndiff SCCA	1	1	2	Carbo weekly	No	0	< 2300	1	0-1 regions	No	No	No		No		17/12/2018	1	0	0	0	1	Low risk
204	Male	55	<60	NonkerUndiff SCCA	2	1	2	Cis weekly	No	0	< 2300	1	0-1 regions	No	No	No		No		13/12/2017	1	1	0	0	2	Low risk
205	Male	58	<60	NonkerDiffSCCA	3	2	3	Carbo weekly	Yes	0	< 2300	4	2-6 regions	No	No	No		No		12/12/2017	1	2	2	0	5	High risk
206	Male	63	≥60	NonkerDiffSCCA	1	2	3	Cis triweekly	No	699	< 2300	6	2-6 regions	No	No	No		No		26/12/2017	1	0	2	0	3	Low risk
207	Female	44	<60	NonkerUndiff SCCA	1	1	2	Cis triweekly	Yes	0	< 2300	2	2-6 regions	No	No	No		No		31/5/2018	0	0	2	0	2	Low risk
208	Male	42	<60	NonkerUndiff SCCA	4	1	4	Cis triweekly	Yes	1179	< 2300	1	0-1 regions	No	No	No		No		9/1/2017	1	2	0	0	3	Low risk
209	Male	56	<60	NonkerUndiff SCCA	3	1	3	Carbo weekly	Yes	0	< 2300	1	0-1 regions	No	No	No		No		11/1/2016	1	2	0	0	3	Low risk
210	Male	28	<60	NonkerDiffSCCA	1	3	4	Cis weekly	Yes	934	< 2300	8	7-13 regions	No	No	Yes	21/1/2019	Yes	26/11/2020	12/3/2018	1	0	3	0	4	Low risk
211	Female	64	≥60	NonkerUndiff SCCA	3	1	3	Cis weekly	Yes	5763	≥ 2300	1	0-1 regions	No	No	No		Yes	8/2/2016	17/12/2012	0	2	0	1	3	Low risk
212	Female	36	<60	NonkerUndiff SCCA	3	2	3	Cis triweekly	Yes	8820	≥ 2300	5	2-6 regions	Yes	No	No		Yes	24/5/2018	17/1/2017	0	2	2	1	5	High risk
213	Female	62	≥60	NonkerDiffSCCA	1	1	2	Cis weekly	Yes	2163	< 2300	3	2-6 regions	No	No	No		No		23/4/2018	0	0	2	0	2	Low risk
214	Male	36	<60	NonkerUndiff SCCA	1	2	3	Cis weekly	Yes	4231	≥ 2300	3	2-6 regions	Yes	No	No		No		23/5/2013	1	0	2	1	4	Low risk
215	Female	49	<60	NonkerDiffSCCA	2	2	3	Cis triweekly	Yes	406800	≥ 2300	3	2-6 regions	Yes	No	No		No		3/4/2017	0	1	2	1	4	Low risk
216	Male	63	≥60	NonkerDiffSCCA	1	1	2	Carbo triweekly	Yes	1323	< 2300	3	2-6 regions	Yes	No	Yes	1/12/2016	Yes	2/9/2018	4/4/2016	1	0	2	0	3	Low risk
217	Male	53	<60	NonkerUndiff SCCA	2	2	3	Carbo triweekly	Yes	17730	≥ 2300	5	2-6 regions	Yes	No	No		No		12/6/2017	1	1	2	1	5	High risk
218	Male	46	<60	NonkerUndiff SCCA	3	2	3	Cis triweekly	Yes	5229	≥ 2300	3	2-6 regions	Yes	No	No		No		25/4/2016	1	2	2	1	6	High risk
219	Male	78	≥60	NonkerDiffSCCA	2	2	3	Carbo weekly	No	1494	< 2300	6	2-6 regions	Yes	Yes	Yes	31/7/2019	No		4/9/2017	1	1	2	0	4	Low risk
220	Male	50	<60	NonkerUndiff SCCA	1	1	2	Cis triweekly	No	0	< 2300	2	2-6 regions	Yes	No	No		No		26/9/2016	1	0	2	0	3	Low risk
221	Female	76	≥60	NonkerUndiff SCCA	3	1	3	Carbo weekly	No	4100	≥ 2300	3	2-6 regions	No	No	No		Yes	22/4/2016	25/5/2015	0	2	2	1	5	High risk
222	Male	66	≥60	NonkerUndiff SCCA	3	0	3	Cis weekly	Yes	0	< 2300	0	0-1 regions	No	No	No		No		5/9/2016	1	2	0	0	3	Low risk
223	Female	38	<60	NonkerUndiff SCCA	3	1	3	Cis triweekly	Yes	572	< 2300	3	2-6 regions	No	No	No		No		9/9/2019	0	2	2	0	4	Low risk
224	Male	60	≥60	NonkerDiffSCCA	3	0	3	Cis weekly	No	53046	≥ 2300	0	0-1 regions	No	No	No		No		26/7/2019	1	2	0	1	4	Low risk
225	Male	48	<60	NonkerUndiff SCCA	3	1	3	Cis weekly	No	14510	≥ 2300	1	0-1 regions	No	No	No		No		22/10/2018	1	2	0	1	4	Low risk
226	Male	31	<60	NonkerUndiff SCCA	2	1	2	Cis triweekly	Yes	22600	≥ 2300	2	2-6 regions	Yes	No	Yes	1/2/2017	Yes	22/7/2018	23/11/2015	1	1	2	1	5	High risk
227	Male	41	<60	NonkerUndiff SCCA	3	1	3	Cis triweekly	No	1613	< 2300	2	2-6 regions	No	No	No		No		26/2/2018	1	2	2	0	5	High risk
228	Male	57	<60	NonkerDiffSCCA	4	0	4	Cis weekly	Yes	4330	≥ 2300	0	0-1 regions	No	No	No		No		23/2/2015	1	2	0	1	4	Low risk
229	Female	60	≥60	NonkerUndiff SCCA	3	2	3	Cis triweekly	Yes	12870	≥ 2300	8	7-13 regions	Yes	No	No		No		4/4/2016	0	2	3	1	6	High risk
230	Male	38	<60	NonkerUndiff SCCA	3	3	4	Cis weekly	Yes	2789	≥ 2300	12	7-13 regions	Yes	Yes	Yes	11/4/2017	Yes	28/10/2017	18/8/2016	1	2	3	1	7	High risk
231	Male	61	≥60	NonkerUndiff SCCA	2	0	2	Cis weekly	No	6742	≥ 2300	0	0-1 regions	No	No	No		No		26/5/2017	1	1	0	1	3	Low risk
232	Male	58	<60	NonkerDiffSCCA	1	1	2	Cis weekly	Yes	0	< 2300	1	0-1 regions	No	No	No		No		28/10/2019	1	0	0	0	1	Low risk
233	Male	52	<60	Basaloid SCCA	1	1	2	Unknown	Yes	0	< 2300	1	0-1 regions	No	No	No		No		16/4/2015	1	0	0	0	1	Low risk
234	Female	52	<60	NonkerUndiff SCCA	1	2	3	Cis triweekly	No	644	< 2300	3	2-6 regions	Yes	No	No		No		30/1/2017	0	0	2	0	2	Low risk
235	Male	42	<60	NonkerUndiff SCCA	1	1	2	Cis weekly	Yes	5483	≥ 2300	2	2-6 regions	No	No	No		No		23/12/2013	1	0	2	1	4	Low risk
236	Male	60	≥60	NonkerUndiff SCCA	1	2	3	Cis weekly	Yes	3860	≥ 2300	5	2-6 regions	Yes	No	No		Yes	4/2/2021	20/5/2015	1	0	2	1	4	Low risk
237	Male	57	<60	NonkerUndiff SCCA	3	1	3	Cis weekly	No	685	< 2300	1	0-1 regions	No	No	No		No		17/1/2017	1	2	0	0	3	Low risk
238	Female	72	≥60	NonkerUndiff SCCA	3	1	3	Carbo weekly	No	3070	≥ 2300	1	0-1 regions	No	No	No		Yes	21/1/2020	20/11/2017	0	2	0	1	3	Low risk
239	Female	53	<60	NonkerUndiff SCCA	1	1	2	Cis weekly	Yes	2640	≥ 2300	3	2-6 regions	No	No	No		No		18/1/2017	0	0	2	1	3	Low risk
240	Male	45	<60	NonkerUndiff SCCA	3	3	4	Cis triweekly	Yes	4527	≥ 2300	5	2-6 regions	Yes	Yes	No		No		6/2/2017	1	2	2	1	6	High risk
241	Male	47	<60	NonkerDiffSCCA	1	3	4	Cis weekly	Yes	2789	≥ 2300	10	7-13 regions	No	No	Yes	22/9/2019	No		27/1/2017	1	0	3	1	5	High risk
242	Male	63	≥60	NonkerUndiff SCCA	3	1	3	Cis weekly	Yes	0	< 2300	1	0-1 regions	No	No	Yes	5/3/2018	Yes	25/7/2019	15/12/2015	1	2	0	0	3	Low risk
243	Female	57	<60	NonkerUndiff SCCA	3	1	3	Unknown	Yes	5421	≥ 2300	1	0-1 regions	No	No	No		No		20/2/2013	0	2	0	1	3	Low risk
244	Female	51	<60	NonkerDiffSCCA	4	2	4	Cis weekly	Yes	9690	≥ 2300	4	2-6 regions	No	No	No		No		9/3/2015	0	2	2	1	5	High risk
245	Male	53	<60	NonkerUndiff SCCA	4	2	4	Cis triweekly	Yes	0	< 2300	3	2-6 regions	No	No	Yes	14/12/2020	Yes	26/5/2021	7/9/2017	1	2	2	0	5	High risk
246	Male	61	≥60	NonkerUndiff SCCA	3	0	3	Cis weekly	Yes	0	< 2300	0	0-1 regions	No	No	No		No		23/2/2016	1	2	0	0	3	Low risk
247	Male	48	<60	NonkerDiffSCCA	3	2	3	Cis weekly	Yes	1422	< 2300	4	2-6 regions	Yes	No	No		No		21/2/2019	1	2	2	0	5	High risk
248	Male	71	≥60	NonkerUndiff SCCA	1	2	3	Carbo weekly	No	84780	≥ 2300	5	2-6 regions	Yes	No	Yes	15/10/2019	Yes	17/6/2021	25/2/2016	1	0	2	1	4	Low risk
249	Male	81	≥60	NonkerUndiff SCCA	4	3	4	Carbo weekly	Yes	22770	≥ 2300	8	7-13 regions	Yes	No	No		Yes	27/2/2017	15/3/2016	1	2	3	1	7	High risk
250	Male	53	<60	NonkerUndiff SCCA	3	1	3	Cis triweekly	Yes	0	< 2300	1	0-1 regions	No	No	No		No		31/3/2017	1	2	0	0	3	Low risk
251	Female	67	≥60	NonkerUndiff SCCA	3	3	4	Cis triweekly	Yes	401	< 2300	3	2-6 regions	Yes	No	No		Yes	30/3/2022	16/3/2016	0	2	2	0	4	Low risk
252	Male	37	<60	NonkerUndiff SCCA	1	3	4	Cis triweekly	Yes	2061	< 2300	6	2-6 regions	Yes	No	No		No		27/2/2017	1	0	2	0	3	Low risk
253	Male	38	<60	NonkerUndiff SCCA	2	1	2	Carbo weekly	Yes	0	< 2300	3	2-6 regions	Yes	No	No		No		25/2/2016	1	1	2	0	4	Low risk
254	Male	49	<60	NonkerUndiff SCCA	3	1	3	Carbo weekly	Yes	0	< 2300	1	0-1 regions	No	No	No		Yes	30/4/2021	17/4/2018	1	2	0	0	3	Low risk
255	Male	59	<60	NonkerUndiff SCCA	4	2	4	Cis triweekly	Yes	66690	≥ 2300	5	2-6 regions	Yes	No	No		Yes	6/3/2017	26/5/2016	1	2	2	1	6	High risk
256	Female	44	<60	NonkerUndiff SCCA	3	1	3	Cis weekly	Yes	39759	≥ 2300	1	0-1 regions	Yes	No	No		No		1/7/2019	0	2	0	1	3	Low risk
257	Female	76	≥60	NonkerUndiff SCCA	3	1	3	Cis weekly	Yes	7782	≥ 2300	1	0-1 regions	No	No	No		Yes	29/5/2016	20/4/2015	0	2	0	1	3	Low risk
258	Male	66	≥60	NonkerUndiff SCCA	2	3	4	Cis weekly	Yes	0	< 2300	7	7-13 regions	No	No	No		No		8/4/2015	1	1	3	0	5	High risk
259	Male	46	<60	NonkerUndiff SCCA	4	1	4	Cis triweekly	Yes	2500	≥ 2300	4	2-6 regions	Yes	No	No		No		30/4/2018	1	2	2	1	6	High risk
260	Male	46	<60	NonkerUndiff SCCA	1	1	2	Cis weekly	No	0	< 2300	1	0-1 regions	No	No	No		No		25/3/2019	1	0	0	0	1	Low risk
261	Female	74	≥60	NonkerUndiff SCCA	1	1	2	Cis weekly	No	0	< 2300	2	2-6 regions	No	No	No		No		5/4/2019	0	0	2	0	2	Low risk
262	Male	60	≥60	NonkerUndiff SCCA	1	1	2	Cis weekly	Yes	1035	< 2300	2	2-6 regions	No	No	No		No		9/3/2016	1	0	2	0	3	Low risk
263	Male	44	<60	NonkerUndiff SCCA	3	2	3	Cis weekly	Yes	1179	< 2300	5	2-6 regions	Yes	No	Yes	25/6/2019	Yes	21/4/2020	18/4/2017	1	2	2	0	5	High risk
264	Male	61	≥60	NonkerUndiff SCCA	3	1	3	Cis weekly	Yes	5580	≥ 2300	2	2-6 regions	No	No	No		No		10/6/2015	1	2	2	1	6	High risk
265	Male	52	<60	NonkerUndiff SCCA	3	2	3	Cis triweekly	Yes	0	< 2300	4	2-6 regions	Yes	No	No		No		12/5/2016	1	2	2	0	5	High risk
266	Male	55	<60	NonkerUndiff SCCA	1	1	2	Cis weekly	Yes	439	< 2300	2	2-6 regions	No	No	No		No		17/4/2019	1	0	2	0	3	Low risk
267	Female	81	≥60	NonkerUndiff SCCA	3	0	3	Carbo weekly	No	0	< 2300	0	0-1 regions	No	No	No		Yes	28/1/2021	27/9/2017	0	2	0	0	2	Low risk
268	Female	31	<60	NonkerUndiff SCCA	3	1	3	Cis weekly	No	0	< 2300	2	2-6 regions	No	No	No		No		13/11/2017	0	2	2	0	4	Low risk
269	Male	37	<60	NonkerUndiff SCCA	1	1	2	Cis triweekly	No	0	< 2300	2	2-6 regions	No	No	No		No		17/4/2019	1	0	2	0	3	Low risk
270	Male	61	≥60	NonkerDiffSCCA	3	1	3	Cis weekly	No	2041	< 2300	2	2-6 regions	Yes	No	No		No		3/4/2018	1	2	2	0	5	High risk
271	Male	38	<60	NonkerUndiff SCCA	1	1	2	Cis weekly	No	0	< 2300	1	0-1 regions	No	No	No		No		9/4/2018	1	0	0	0	1	Low risk
272	Male	45	<60	NonkerUndiff SCCA	1	1	2	Cis triweekly	No	0	< 2300	1	0-1 regions	No	No	No		No		9/4/2018	1	0	0	0	1	Low risk
273	Male	45	<60	NonkerDiffSCCA	3	2	3	Cis triweekly	Yes	14670	≥ 2300	2	2-6 regions	No	No	Yes	15/5/2018	Yes	4/5/2020	24/5/2017	1	2	2	1	6	High risk
274	Female	55	<60	NonkerDiffSCCA	1	2	3	Cis weekly	Yes	0	< 2300	4	2-6 regions	Yes	No	No		No		24/8/2015	0	0	2	0	2	Low risk
275	Male	33	<60	NonkerUndiff SCCA	1	1	2	Cis triweekly	Yes	1269	< 2300	2	2-6 regions	Yes	No	No		No		1/6/2017	1	0	2	0	3	Low risk
276	Female	38	<60	NonkerUndiff SCCA	1	1	2	Cis triweekly	Yes	0	< 2300	1	0-1 regions	Yes	No	No		No		25/8/2016	0	0	0	0	0	Low risk
277	Female	53	<60	NonkerUndiff SCCA	1	1	2	Cis weekly	No	7413	≥ 2300	1	0-1 regions	No	No	No		No		15/6/2016	0	0	0	1	1	Low risk
278	Male	57	<60	NonkerUndiff SCCA	4	2	4	Carbo weekly	Yes	0	< 2300	9	7-13 regions	Yes	No	Yes	6/5/2017	Yes	6/12/2017	9/6/2016	1	2	3	0	6	High risk
279	Female	72	≥60	NonkerUndiff SCCA	3	1	3	Carbo weekly	Yes	685	< 2300	1	0-1 regions	No	No	No		Yes	13/10/2020	21/7/2016	0	2	0	0	2	Low risk
280	Male	57	<60	NonkerDiffSCCA	1	1	2	Carbo triweekly	Yes	0	< 2300	3	2-6 regions	Yes	No	No		No		23/1/2017	1	0	2	0	3	Low risk
281	Male	73	≥60	NonkerDiffSCCA	2	1	2	Carbo triweekly	No	39600	≥ 2300	2	2-6 regions	Yes	No	No		Yes	19/10/2017	8/5/2017	1	1	2	1	5	High risk
282	Male	69	≥60	NonkerUndiff SCCA	3	1	3	Carbo triweekly	No	0	< 2300	1	0-1 regions	No	No	No		No		21/11/2016	1	2	0	0	3	Low risk
283	Male	59	<60	NonkerUndiff SCCA	1	1	2	Cis weekly	Yes	0	< 2300	1	0-1 regions	Yes	No	No		No		26/6/2017	1	0	0	0	1	Low risk
284	Male	52	<60	NonkerDiffSCCA	2	3	4	Cis weekly	Yes	5640	≥ 2300	6	2-6 regions	Yes	No	No		No		23/6/2015	1	1	2	1	5	High risk
285	Male	63	≥60	NonkerUndiff SCCA	3	1	3	Cis weekly	Yes	1710	< 2300	2	2-6 regions	Yes	No	No		No		21/7/2016	1	2	2	0	5	High risk
286	Male	57	<60	NonkerUndiff SCCA	3	2	3	Cis weekly	Yes	264000	≥ 2300	7	7-13 regions	Yes	No	Yes	27/9/2018	Yes	28/9/2021	15/6/2015	1	2	3	1	7	High risk
287	Female	60	≥60	NonkerUndiff SCCA	1	2	3	Cis weekly	Yes	0	< 2300	3	2-6 regions	Yes	No	No		No		29/5/2019	0	0	2	0	2	Low risk
288	Male	56	<60	NonkerUndiff SCCA	4	3	4	Cis weekly	Yes	59040	≥ 2300	7	7-13 regions	Yes	Yes	Yes	13/2/2018	Yes	9/7/2018	13/7/2017	1	2	3	1	7	High risk
289	Female	42	<60	NonkerDiffSCCA	3	1	3	Cis weekly	Yes	0	< 2300	2	2-6 regions	Yes	No	No		No		23/6/2016	0	2	2	0	4	Low risk
290	Male	41	<60	NonkerUndiff SCCA	1	1	2	Cis triweekly	Yes	408	< 2300	3	2-6 regions	No	No	No		No		26/6/2017	1	0	2	0	3	Low risk
291	Male	38	<60	NonkerUndiff SCCA	3	1	3	Carbo triweekly	Yes	0	< 2300	3	2-6 regions	Yes	No	No		No		19/6/2017	1	2	2	0	5	High risk
292	Male	53	<60	NonkerUndiff SCCA	3	0	3	Carbo weekly	Yes	5850	≥ 2300	0	0-1 regions	No	No	No		No		21/7/2016	1	2	0	1	4	Low risk
293	Female	36	<60	NonkerUndiff SCCA	3	1	3	Cis weekly	Yes	0	< 2300	1	0-1 regions	No	No	No		No		30/6/2016	0	2	0	0	2	Low risk
294	Female	46	<60	NonkerUndiff SCCA	1	1	2	Cis weekly	Yes	0	< 2300	1	0-1 regions	No	No	No		No		19/6/2017	0	0	0	0	0	Low risk
295	Male	62	≥60	NonkerUndiff SCCA	1	1	2	Carbo triweekly	Yes	2538	≥ 2300	5	2-6 regions	Yes	No	No		No		30/6/2016	1	0	2	1	4	Low risk
296	Male	60	≥60	NonkerUndiff SCCA	1	2	3	Carbo weekly	Yes	5542	≥ 2300	10	7-13 regions	Yes	No	Yes	5/6/2018	Yes	9/10/2018	12/7/2016	1	0	3	1	5	High risk
297	Male	66	≥60	NonkerDiffSCCA	2	3	4	Cis weekly	Yes	2397	≥ 2300	10	7-13 regions	No	No	Yes	3/3/2014	Yes	18/4/2014	8/6/2013	1	1	3	1	6	High risk
298	Male	52	<60	NonkerUndiff SCCA	3	1	3	Cis triweekly	Yes	353	< 2300	1	0-1 regions	No	No	No		No		17/7/2016	1	2	0	0	3	Low risk
299	Male	21	<60	NonkerUndiff SCCA	2	2	3	Cis triweekly	Yes	40704	≥ 2300	6	2-6 regions	Yes	No	Yes	5/2/2020	No		18/7/2018	1	1	2	1	5	High risk
300	Male	49	<60	NonkerUndiff SCCA	1	1	2	Cis weekly	Yes	7721	≥ 2300	1	0-1 regions	No	No	No		No		5/7/2016	1	0	0	1	2	Low risk
301	Male	54	<60	NonkerUndiff SCCA	1	1	2	Cis weekly	Yes	8413	≥ 2300	1	0-1 regions	No	No	No		No		17/1/2014	1	0	0	1	2	Low risk
302	Male	32	<60	NonkerDiffSCCA	2	0	2	Cis weekly	Yes	0	< 2300	0	0-1 regions	No	No	No		No		19/6/2018	1	1	0	0	2	Low risk
303	Male	65	≥60	NonkerUndiff SCCA	4	2	4	Carbo weekly	Yes	5572	≥ 2300	2	2-6 regions	Yes	No	No		No		10/1/2018	1	2	2	1	6	High risk
304	Female	75	≥60	NonkerUndiff SCCA	3	1	3	Carbo weekly	No	4837	≥ 2300	1	0-1 regions	Yes	No	No		Yes	7/8/2019	11/3/2019	0	2	0	1	3	Low risk
305	Male	39	<60	NonkerUndiff SCCA	3	1	3	Cis weekly	Yes	4467	≥ 2300	1	0-1 regions	Yes	No	No		No		29/11/2013	1	2	0	1	4	Low risk
306	Female	57	<60	NonkerDiffSCCA	1	1	2	Cis triweekly	Yes	13860	≥ 2300	3	2-6 regions	Yes	No	No		No		22/8/2016	0	0	2	1	3	Low risk
307	Male	22	<60	NonkerUndiff SCCA	1	1	2	Cis triweekly	Yes	354	< 2300	1	0-1 regions	No	No	No		No		19/7/2018	1	0	0	0	1	Low risk
308	Male	48	<60	NonkerUndiff SCCA	2	0	2	Cis weekly	Yes	8965	≥ 2300	0	0-1 regions	No	No	No		No		22/11/2013	1	1	0	1	3	Low risk
309	Male	51	<60	NonkerDiffSCCA	3	1	3	Cis weekly	Yes	0	< 2300	2	2-6 regions	No	No	No		No		24/8/2015	1	2	2	0	5	High risk
310	Male	55	<60	NonkerDiffSCCA	2	1	2	Cis weekly	Yes	0	< 2300	2	2-6 regions	No	No	No		No		31/8/2016	1	1	2	0	4	Low risk
311	Male	41	<60	NonkerUndiff SCCA	3	3	4	Cis weekly	Yes	37080	≥ 2300	7	7-13 regions	Yes	No	Yes	21/11/2018	Yes	24/5/2019	31/8/2017	1	2	3	1	7	High risk
312	Male	45	<60	NonkerUndiff SCCA	3	1	3	Cis triweekly	No	1416	< 2300	1	0-1 regions	Yes	No	Yes	28/1/2019	Yes	13/10/2019	14/8/2018	1	2	0	0	3	Low risk
313	Male	59	<60	NonkerUndiff SCCA	1	2	3	Cis weekly	Yes	0	< 2300	4	2-6 regions	No	No	No		No		16/9/2019	1	0	2	0	3	Low risk
314	Female	64	≥60	NonkerUndiff SCCA	4	1	4	Cis triweekly	Yes	770	< 2300	2	2-6 regions	Yes	No	No		No		20/4/2016	0	2	2	0	4	Low risk
315	Female	40	<60	NonkerDiffSCCA	3	3	4	Cis triweekly	No	1422	< 2300	11	7-13 regions	Yes	No	Yes	4/12/2017	Yes	11/1/2020	8/9/2016	0	2	3	0	5	High risk
316	Female	61	≥60	NonkerDiffSCCA	1	1	2	Cis weekly	No	2488	≥ 2300	1	0-1 regions	No	No	No		No		10/8/2017	0	0	0	1	1	Low risk
317	Male	56	<60	NonkerUndiff SCCA	2	2	3	Cis triweekly	Yes	347	< 2300	4	2-6 regions	No	No	No		No		30/8/2017	1	1	2	0	4	Low risk
318	Female	23	<60	NonkerDiffSCCA	3	1	3	Cis weekly	Yes	0	< 2300	1	0-1 regions	Yes	No	No		No		20/9/2016	0	2	0	0	2	Low risk
319	Male	71	≥60	NonkerDiffSCCA	2	1	2	Carbo weekly	Yes	2672	≥ 2300	3	2-6 regions	No	No	Yes	16/8/2016	Yes	18/8/2018	21/9/2015	1	1	2	1	5	High risk
320	Female	60	≥60	NonkerUndiff SCCA	1	1	2	Cis weekly	Yes	1080	< 2300	1	0-1 regions	Yes	No	No		No		27/1/2016	0	0	0	0	0	Low risk
321	Male	45	<60	NonkerUndiff SCCA	4	2	4	Cis triweekly	Yes	42000	≥ 2300	3	2-6 regions	Yes	No	Yes	29/8/2018	No		19/9/2016	1	2	2	1	6	High risk
322	Male	41	<60	NonkerUndiff SCCA	3	3	4	Cis triweekly	Yes	7832	≥ 2300	8	7-13 regions	No	Yes	Yes	26/1/2016	Yes	1/6/2017	4/11/2013	1	2	3	1	7	High risk
323	Female	32	<60	NonkerDiffSCCA	2	3	4	Cis triweekly	Yes	846	< 2300	6	2-6 regions	Yes	No	No		No		25/9/2017	0	1	2	0	3	Low risk
324	Male	22	<60	NonkerUndiff SCCA	4	2	4	Cis triweekly	Yes	4152	≥ 2300	3	2-6 regions	No	No	No		No		24/10/2017	1	2	2	1	6	High risk
325	Male	31	<60	NonkerUndiff SCCA	1	1	2	Cis triweekly	Yes	5674	≥ 2300	3	2-6 regions	Yes	No	No		No		31/8/2017	1	0	2	1	4	Low risk
326	Male	34	<60	NonkerUndiff SCCA	3	1	3	Cis weekly	No	0	< 2300	1	0-1 regions	No	No	No		No		26/9/2016	1	2	0	0	3	Low risk
327	Male	59	<60	NonkerUndiff SCCA	2	2	3	Cis triweekly	Yes	0	< 2300	3	2-6 regions	No	No	No		No		4/10/2017	1	1	2	0	4	Low risk
328	Female	40	<60	NonkerUndiff SCCA	2	0	2	Cis weekly	No	0	< 2300	0	0-1 regions	No	No	No		No		24/10/2017	0	1	0	0	1	Low risk
329	Male	55	<60	NonkerUndiff SCCA	3	1	3	Cis weekly	Yes	0	< 2300	1	0-1 regions	No	No	No		No		28/9/2015	1	2	0	0	3	Low risk
330	Male	29	<60	NonkerUndiff SCCA	1	1	2	Cis triweekly	Yes	0	< 2300	1	0-1 regions	No	No	No		No		3/10/2016	1	0	0	0	1	Low risk
331	Male	48	<60	NonkerUndiff SCCA	1	2	3	Cis weekly	Yes	0	< 2300	5	2-6 regions	Yes	No	No		No		3/11/2015	1	0	2	0	3	Low risk
332	Male	33	<60	NonkerUndiff SCCA	2	1	2	Cis weekly	Yes	0	< 2300	1	0-1 regions	No	No	No		No		12/10/2015	1	1	0	0	2	Low risk
333	Female	49	<60	NonkerUndiff SCCA	1	1	2	Cis triweekly	Yes	0	< 2300	4	2-6 regions	No	No	No		No		5/10/2017	0	0	2	0	2	Low risk
334	Male	46	<60	NonkerUndiff SCCA	1	3	4	Cis triweekly	Yes	3430	≥ 2300	5	2-6 regions	Yes	No	No		No		18/10/2017	1	0	2	1	4	Low risk
335	Male	51	<60	NonkerUndiff SCCA	3	3	4	Cis triweekly	Yes	157000	≥ 2300	10	7-13 regions	Yes	No	No		No		26/10/2015	1	2	3	1	7	High risk
336	Male	46	<60	NonkerUndiff SCCA	4	1	4	Cis triweekly	Yes	987	< 2300	2	2-6 regions	Yes	No	Yes	1/1/2019	Yes	20/11/2019	24/10/2017	1	2	2	0	5	High risk
337	Male	59	<60	NonkerUndiff SCCA	3	1	3	Carbo weekly	No	7998	≥ 2300	1	0-1 regions	No	No	No		No		30/5/2018	1	2	0	1	4	Low risk

338	Female	26	<60	NonkerUndiff SCCA	3	1	3	Cis_triweekly	Yes	0	< 2300	1	0-1 regions	No	No	No		No		20/11/2017	0	2	0	0	2	Low_risk
339	Male	42	<60	NonkerUndiff SCCA	3	2	3	Cis_weekly	Yes	149400	≥ 2300	7	7-13 regions	Yes	No	No		Yes	18/5/2019	16/11/2016	1	2	3	1	7	High_risk
340	Male	64	≥60	NonkerUndiff SCCA	3	1	3	Carbo_weekly	Yes	2061	< 2300	1	0-1 regions	Yes	No	No		Yes	21/2/2021	8/5/2017	1	2	0	0	3	Low_risk
341	Female	25	<60	NonkerUndiff SCCA	1	3	4	Cis_triweekly	Yes	3564	≥ 2300	2	2-6 regions	Yes	No	No		No		21/11/2016	0	0	2	1	3	Low_risk
342	Male	31	<60	NonkerUndiff SCCA	3	1	3	Carbo_triweekly	Yes	1296	< 2300	4	2-6 regions	No	No	Yes	23/1/2020	Yes	27/5/2020	15/11/2018	1	2	2	0	5	High_risk
343	Male	53	<60	NonkerDiffSCCA	1	1	2	Cis_weekly	Yes	6130	≥ 2300	2	2-6 regions	No	No	No		No		6/11/2015	1	0	2	1	4	Low_risk
344	Male	64	≥60	NonkerUndiff SCCA	3	0	3	Carbo_weekly	Yes	0	< 2300	0	0-1 regions	No	No	No		No		22/10/2018	1	2	0	0	3	Low_risk
345	Male	51	<60	NonkerUndiff SCCA	3	3	4	Cis_weekly	Yes	162000	≥ 2300	13	7-13 regions	No	No	No		No		17/11/2016	1	2	3	1	7	High_risk
346	Male	53	<60	NonkerUndiff SCCA	4	1	4	Unknown	Unknown	6692	≥ 2300	2	2-6 regions	Yes	No	No		Yes	15/10/2017	22/12/2015	1	2	2	1	6	High_risk
347	Male	54	<60	NonkerUndiff SCCA	1	1	2	Cis_triweekly	No	7153	≥ 2300	2	2-6 regions	No	No	Yes	8/10/2020	Yes	16/1/2022	3/10/2019	1	0	2	1	4	Low_risk
348	Male	33	<60	NonkerDiffSCCA	2	0	2	Cis_weekly	No	0	< 2300	0	0-1 regions	No	No	No		No		14/11/2018	1	1	0	0	2	Low_risk
349	Female	26	<60	NonkerUndiff SCCA	3	2	3	Cis_triweekly	No	516	< 2300	3	2-6 regions	No	No	No		No		15/11/2018	0	2	2	0	4	Low_risk
350	Female	52	<60	NonkerUndiff SCCA	1	1	2	Cis_triweekly	Yes	894	< 2300	2	2-6 regions	No	No	No		Yes	29/3/2019	25/12/2017	0	0	2	0	2	Low_risk
351	Female	52	<60	NonkerDiffSCCA	4	1	4	Cis_weekly	No	0	< 2300	3	2-6 regions	Yes	No	No		No		13/11/2018	0	2	2	0	4	Low_risk
352	Male	45	<60	NonkerUndiff SCCA	3	1	3	Cis_weekly	No	762	< 2300	2	2-6 regions	No	No	No		No		6/12/2017	1	2	2	0	5	High_risk
353	Male	59	<60	NonkerUndiff SCCA	1	1	2	Carbo_weekly	No	0	< 2300	1	0-1 regions	No	No	No		No		23/6/2015	1	0	0	0	1	Low_risk
354	Male	51	<60	NonkerUndiff SCCA	1	1	2	Cis_weekly	No	0	< 2300	2	2-6 regions	No	No	No		No		24/6/2019	1	0	2	0	3	Low_risk
355	Male	52	<60	NonkerUndiff SCCA	3	1	3	Cis_weekly	No	0	< 2300	1	0-1 regions	No	No	No		No		25/6/2018	1	2	0	0	3	Low_risk
356	Female	59	<60	NonkerUndiff SCCA	2	1	2	Cis_weekly	No	0	< 2300	2	2-6 regions	No	No	No		No		14/1/2019	0	1	2	0	3	Low_risk
357	Male	58	<60	NonkerDiffSCCA	4	1	4	Cis_weekly	Yes	0	< 2300	1	0-1 regions	Yes	No	No		No		13/2/2018	1	2	0	0	3	Low_risk
358	Female	28	<60	NonkerUndiff SCCA	4	1	4	Cis_triweekly	Yes	2339	≥ 2300	1	0-1 regions	No	No	No		No		18/3/2019	0	2	0	1	3	Low_risk
359	Male	67	≥60	NonkerUndiff SCCA	3	3	4	Carbo_weekly	No	6070	≥ 2300	6	2-6 regions	Yes	No	Yes	17/1/2016	No		23/3/2015	1	2	2	1	6	High_risk
360	Male	48	<60	NonkerDiffSCCA	3	2	3	Unknown	Yes	5170	≥ 2300	5	2-6 regions	No	No	Yes	8/11/2015	Yes	6/5/2016	30/3/2015	1	2	2	1	6	High_risk
361	Male	55	<60	NonkerUndiff SCCA	4	1	4	Carbo_triweekly	Yes	142689	≥ 2300	3	2-6 regions	No	No	Yes	1/7/2019	No		12/3/2018	1	2	2	1	6	High_risk
362	Male	52	<60	NonkerUndiff SCCA	4	1	4	Carbo_triweekly	Yes	1775	< 2300	4	2-6 regions	Yes	No	No		No		2/4/2018	1	2	2	0	5	High_risk
363	Male	38	<60	NonkerUndiff SCCA	1	1	2	Unknown	Yes	3427	≥ 2300	3	2-6 regions	No	No	No		No		12/3/2018	1	0	2	1	4	Low_risk
364	Female	35	<60	NonkerUndiff SCCA	4	2	4	Carbo_weekly	No	1054	< 2300	6	2-6 regions	No	No	No		No		14/1/2019	0	2	2	0	4	Low_risk
365	Female	65	≥60	NonkerUndiff SCCA	1	3	4	Cis_triweekly	Yes	12780	≥ 2300	5	2-6 regions	Yes	Yes	No		No		30/5/2016	0	0	2	1	3	Low_risk
366	Male	28	<60	NonkerUndiff SCCA	1	2	3	Cis_weekly	Yes	12363	≥ 2300	5	2-6 regions	Yes	No	No		No		9/5/2018	1	0	2	1	4	Low_risk
367	Male	70	≥60	NonkerUndiff SCCA	4	1	4	Carbo/pac_wee	Yes	3726	≥ 2300	2	2-6 regions	Yes	No	Yes	7/6/2018	Yes	28/7/2019	6/6/2017	1	2	2	1	6	High_risk
368	Male	27	<60	NonkerUndiff SCCA	3	3	4	Cis_triweekly	Yes	6490	≥ 2300	6	2-6 regions	No	No	Yes	1/11/2015	Yes	5/3/2016	15/6/2015	1	2	2	1	6	High_risk
369	Male	39	<60	NonkerUndiff SCCA	4	0	4	Cis_weekly	Yes	0	< 2300	0	0-1 regions	No	No	No		Yes	15/10/2018	1/8/2015	1	2	0	0	3	Low_risk
370	Male	59	<60	NonkerUndiff SCCA	4	3	4	Cis_triweekly	Yes	34328	≥ 2300	6	2-6 regions	Yes	Yes	No		Yes	28/4/2020	11/6/2018	1	2	2	1	6	High_risk
371	Male	34	<60	NonkerUndiff SCCA	4	2	4	Cis_triweekly	No	340	< 2300	3	2-6 regions	No	No	Yes	23/3/2020	No		11/6/2018	1	2	2	0	5	High_risk
372	Female	64	≥60	NonkerUndiff SCCA	1	2	3	Carbo_weekly	Yes	72600	≥ 2300	4	2-6 regions	Yes	No	No		Yes	2/11/2021	9/9/2015	0	0	2	1	3	Low_risk
373	Female	46	<60	NonkerUndiff SCCA	4	2	4	Carbo_triweekly	No	508	< 2300	3	2-6 regions	Yes	No	No		No		31/7/2018	0	2	2	0	4	Low_risk
374	Male	35	<60	NonkerUndiff SCCA	4	3	4	Cis_triweekly	Yes	6600	≥ 2300	7	7-13 regions	No	No	No		No		25/10/2016	1	2	3	1	7	High_risk
375	Female	65	≥60	NonkerUndiff SCCA	3	1	3	Carbo/pac_wee	No	0	< 2300	1	0-1 regions	No	No	No		No		14/8/2018	0	2	0	0	2	Low_risk
376	Male	35	<60	NonkerDiffSCCA	4	1	4	Cis_triweekly	Yes	8900	≥ 2300	3	2-6 regions	No	No	No		No		19/10/2015	1	2	2	1	6	High_risk
377	Female	60	≥60	NonkerUndiff SCCA	4	1	4	Cis_triweekly	Yes	704	< 2300	1	0-1 regions	No	No	No		No		30/10/2017	0	2	0	0	2	Low_risk
378	Male	61	≥60	NonkerUndiff SCCA	4	1	4	Cis_weekly	No	0	< 2300	2	2-6 regions	No	No	Yes	10/8/2017	Yes	4/9/2017	28/12/2015	1	2	2	0	5	High_risk
379	Male	66	≥60	NonkerDiffSCCA	4	2	4	Cis_triweekly	Yes	20520	≥ 2300	6	2-6 regions	Yes	Yes	No		Yes	28/1/2021	16/11/2016	1	2	2	1	6	High_risk
380	Male	40	<60	NonkerUndiff SCCA	3	3	4	Cis_triweekly	Yes	5612	≥ 2300	5	2-6 regions	Yes	No	No		No		2/5/2018	1	2	2	1	6	High_risk
381	Male	45	<60	NonkerUndiff SCCA	4	3	4	Cis_triweekly	Yes	5139	≥ 2300	13	7-13 regions	Yes	No	No		No		28/12/2015	1	2	3	1	7	High_risk
382	Male	40	<60	NonkerDiffSCCA	4	1	4	Cis_triweekly	Yes	1125	< 2300	3	2-6 regions	Yes	No	Yes	9/8/2017	Yes	22/4/2018	11/1/2017	1	2	2	0	5	High_risk
383	Male	44	<60	NonkerDiffSCCA	4	2	4	Cis_triweekly	Yes	71624	≥ 2300	6	2-6 regions	Yes	No	No		No		12/2/2018	1	2	2	1	6	High_risk
384	Female	37	<60	NonkerUndiff SCCA	3	2	3	Cis_triweekly	Yes	0	< 2300	5	2-6 regions	Yes	Yes	No		No		10/3/2015	0	2	2	0	4	Low_risk
385	Female	25	<60	NonkerUndiff SCCA	1	1	2	Carbo_triweekly	No	0	< 2300	2	2-6 regions	No	No	No		No		1/6/2017	0	0	2	0	2	Low_risk
386	Female	38	<60	NonkerUndiff SCCA	3	3	4	Cis_weekly	Yes	0	< 2300	4	2-6 regions	Yes	No	No		No		20/11/2017	0	2	2	0	4	Low_risk
387	Male	43	<60	NonkerUndiff SCCA	2	2	3	Cis_weekly	Yes	2674	≥ 2300	5	2-6 regions	Yes	No	No		No		20/11/2017	1	1	2	1	5	High_risk
388	Female	54	<60	NonkerDiffSCCA	4	2	4	Cis_triweekly	Yes	0	< 2300	3	2-6 regions	No	No	No		No		29/10/2015	0	2	2	0	4	Low_risk
389	Female	37	<60	NonkerUndiff SCCA	3	3	4	Carbo_weekly	No	1161	< 2300	10	7-13 regions	Yes	No	No		No		3/5/2017	0	2	3	0	5	High_risk
390	Male	71	≥60	NonkerUndiff SCCA	3	2	3	Carbo_weekly	No	2280	< 2300	3	2-6 regions	Yes	No	No		Yes	7/6/2020	28/11/2016	1	2	2	0	5	High_risk
391	Male	43	<60	NonkerUndiff SCCA	2	2	3	Cis_weekly	Yes	50517	≥ 2300	7	7-13 regions	Yes	Yes	No		No		28/5/2019	1	1	3	1	6	High_risk
392	Male	39	<60	NonkerUndiff SCCA	1	1	2	Cis_weekly	Yes	0	< 2300	1	0-1 regions	No	No	No		No		15/12/2014	1	0	0	0	1	Low_risk
393	Female	52	<60	NonkerDiffSCCA	1	1	2	Cis_triweekly	Yes	0	< 2300	2	2-6 regions	No	No	No		No		30/10/2017	0	0	2	0	2	Low_risk
