# Supplementary figures and images for: Development and validation of a practical score to predict 3-year distant metastatic free survival in nasopharyngeal carcinoma incorporating the number of lymph node regions
Source: PLoS One. 2024 Aug 27;19(8):e0309436. doi: 10.1371/journal.pone.0309436 (PMC11349101; doi:10.1371/journal.pone.0309436)

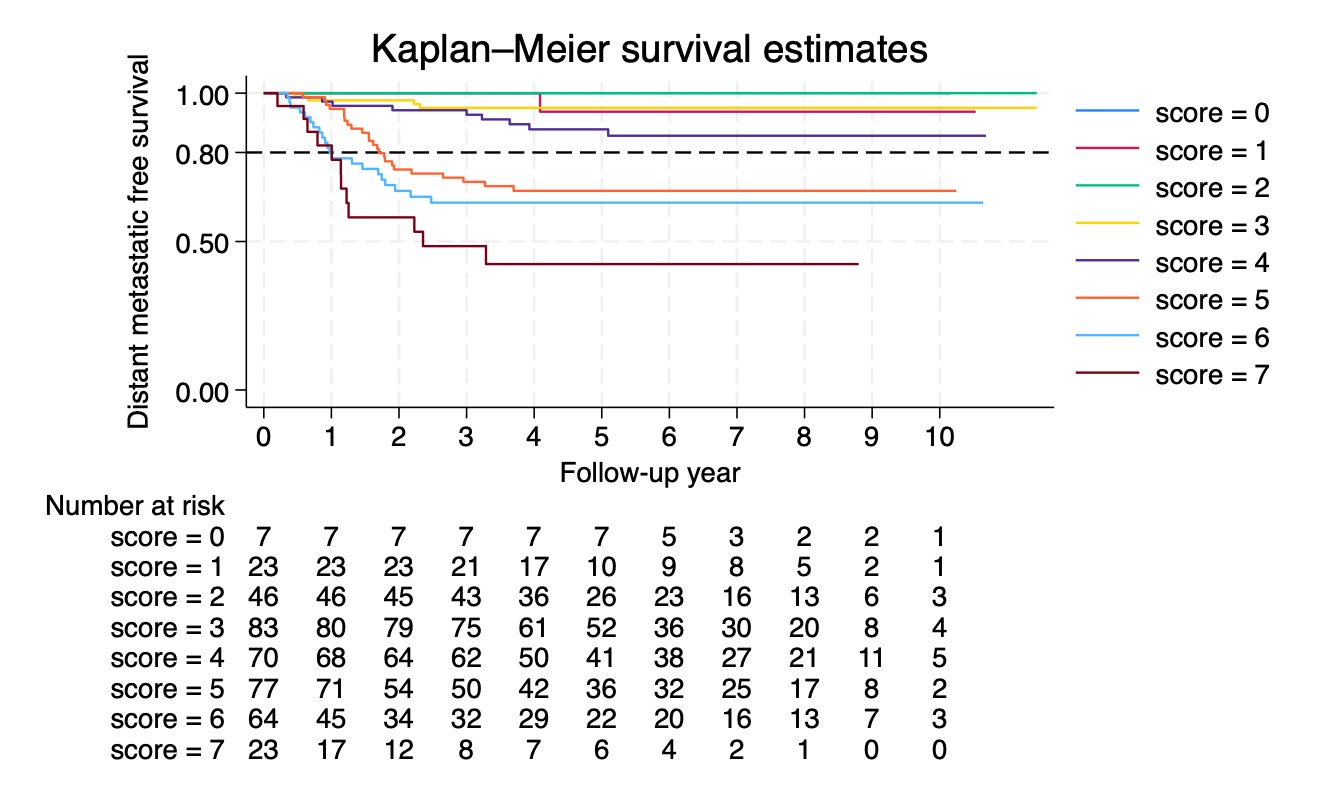

Supplement: S1 Fig — (TIF) [file pone.0309436.s003.tif]

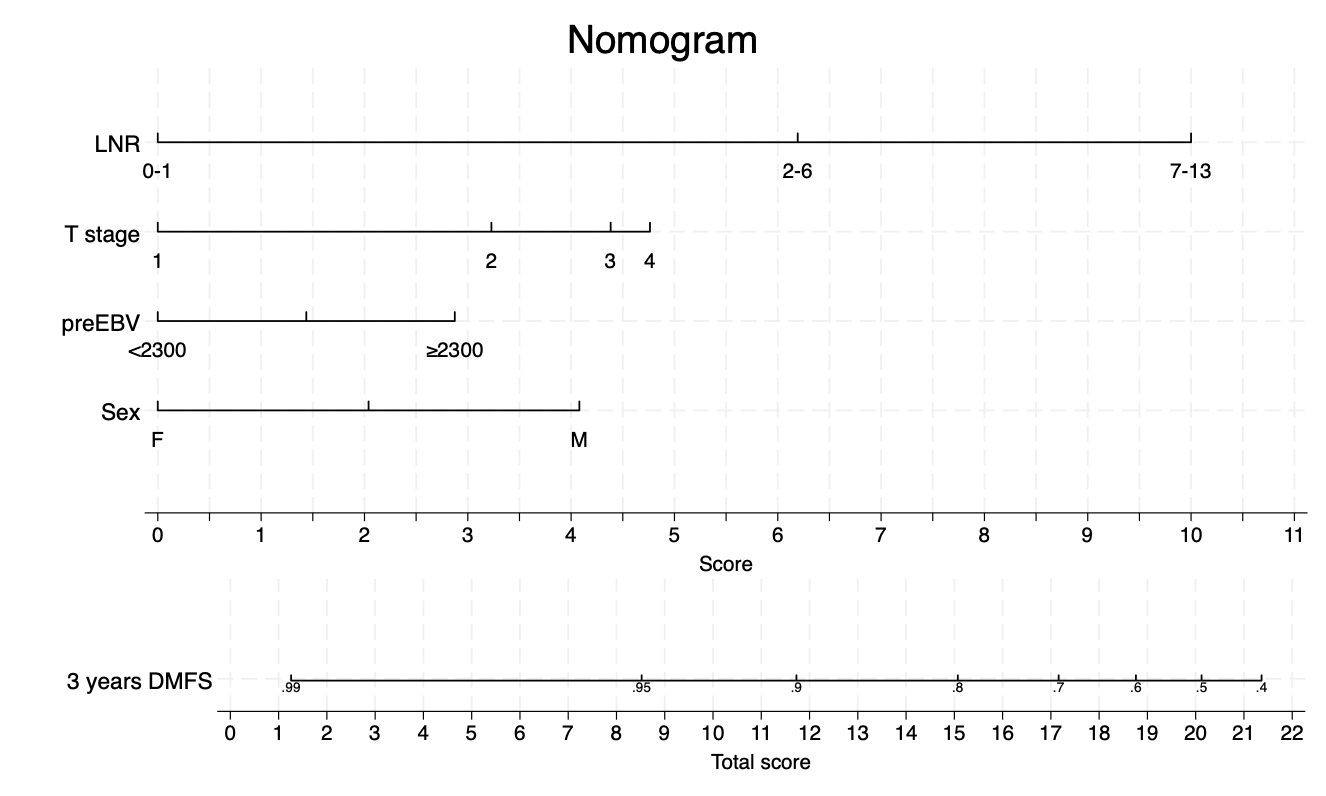

Supplement: S2 Fig — (TIF) [file pone.0309436.s004.tif]
