# Supplementary material for: Development and validation of a practical score to predict 3-year distant metastatic free survival in nasopharyngeal carcinoma incorporating the number of lymph node regions
Source: PLoS One. 2024 Aug 27;19(8):e0309436. doi: 10.1371/journal.pone.0309436 (PMC11349101; doi:10.1371/journal.pone.0309436)
Supplement: Attachment — Submitted filename: Response to reviewers.docx [file pone.0309436.s005.docx]

Professor Lu Zhang

Academic Editor

*PLOS ONE* Journal

August 1, 2024

Dear Professor Lu Zhang:

Regarding the manuscript ID PONE-D-24-23002 entitled “**Development and validation of a practical score to predict 3-year distant metastatic free survival in nasopharyngeal carcinoma incorporating the number of lymph node regions.**”

Thank you very much for carefully reviewing our manuscript and giving us very useful comments and the opportunity to revise. We have made changes in the manuscript according to the suggestions as the followings:

**Editor**

1. Please ensure that your manuscript meets PLOS ONE's style requirements, including those for file naming.

A1: We have checked PLOS ONE's style requirements as you suggested.

2. We note that you have indicated that there are restrictions to data sharing for this study. For studies involving human research participant data or other sensitive data, we encourage authors to share de-identified or anonymized data.

A2: We have provided all the minimal anonymized data sets necessary to replicate our study findings in the supplementary file (S1 Data) and changed the data sharing statement to: “All relevant data are within the manuscript and its supporting information files.”

3. The ORCiD iD has been validated.

**Reviewer #1**

Q1: The sample size is good for this research work. But this work is confined only with deriving a range of scores. A complete nomogram with such a good sample may give a clear understanding of the predictive powers of the 8 candidates. For improving the writing context, author can consider revising the line 304 of Discussion section.

A1: We appreciate your suggestion. As recommended, we have created a nomogram based on the final predictors and included it in the supplementary file. We initially selected 8 potential candidate predictors, but only the final four predictors were used in our final model We chose not to include the nomogram in the main manuscript to avoid potentially confusing readers when interpreting the simplified score. We have added the following sentence to the result section “We also present our final model as a nomogram to predict 3-year DMFS, providing an option for clinicians to use (S2 Fig).” Please see page 16, lines 229-230.

We have also revised line 304 in the discussion section to enhance clarity.

All the changes are tracked in the revised manuscript with track changes. A clean manuscript (with no track changes) is also provided. We hope that it now meets all the requirements of the journal. We appreciate the opportunity to contribute to the *PLOS ONE* journal.

Sincerely yours,

Anussara Prayongrat, MD, PhD

Corresponding Author
